# Neoadjuvant immunotherapy for locally advanced resectable gastric and gastroesophageal junction adenocarcinoma: a systematic review and meta-analysis

**DOI:** 10.1186/s12893-026-04048-y

**Published:** 2026-07-29

**Authors:** Ifrah Mohamud Osman, Siyu Peng, Heshi Liu, Wang Zhen, Shengjie Ma, Quan Wang

**Affiliations:** Department of Gastrocolorectal Surgery, General Surgery Center, The First Hospital of Jilin University, Changchun, China

**Keywords:** Gastric cancer, Gastroesophageal junction adenocarcinoma, Neoadjuvant immunotherapy, Immune checkpoint inhibitors, Meta-analysis

## Abstract

**Background and objective:**

Neoadjuvant and perioperative immune checkpoint inhibitor (ICI)-based strategies are increasingly being investigated in locally advanced resectable gastric and gastroesophageal junction (GEJ) adenocarcinoma, but the overall efficacy and safety of these approaches remain uncertain. This systematic review and meta-analysis aimed to evaluate pathological response, surgical outcomes, radiological response, and treatment-related safety of ICI-containing preoperative regimens in this setting.

**Methods:**

A systematic search of PubMed/MEDLINE, Embase, Cochrane CENTRAL, Web of Science, and Scopus was conducted from 2014 to 2026. Google Scholar was also searched as a supplementary source for gray literature and citation tracking. Studies evaluating neoadjuvant or perioperative ICI-based therapy in adults with locally advanced resectable gastric or GEJ adenocarcinoma were included. Pooled estimates for pathologic complete response (pCR), major pathologic response (MPR), objective response rate (ORR), R0 resection, and safety outcomes were calculated using random-effects models.

**Results:**

Twenty studies were included. The pooled pCR rate was 22.8% (95% c.i. 18.6% to 27.6%), MPR was 49.5% (95% c.i. 41.8% to 57.1%), ORR was 60.6% (95% c.i. 42.0% to 76.6%), and R0 resection was 97.2% (95% c.i. 94.0% to 98.7%). The pooled rates of grade 3 or higher adverse events, immune-related adverse events, and postoperative surgical complications were 24.0% (95% c.i. 15.6% to 35.2%), 23.2% (95% c.i. 15.4% to 33.5%), and 26.2% (95% c.i. 19.1% to 34.7%), respectively.

**Conclusion:**

Neoadjuvant or perioperative ICI-based therapy is associated with encouraging pathological response and high R0 resection rates in locally advanced resectable gastric and GEJ adenocarcinoma. However, the available evidence is limited by substantial clinical heterogeneity and immature survival data. Larger randomized studies with longer follow-up are needed to confirm long-term oncologic benefit and better define optimal patient selection.

**Supplementary Information:**

The online version contains supplementary material available at https://doi.org/10.1186/s12893-026-04048-y.

## Introduction

 Gastric and gastroesophageal junction (GEJ) adenocarcinomas remain major contributors to global cancer-related morbidity and mortality [1, 2]. In patients with locally advanced resectable disease, surgery remains the foundation of curative-intent treatment; however, surgery alone is frequently inadequate because of nodal involvement, occult micrometastatic disease, and a persistent risk of recurrence. Accordingly, multimodality treatment has become standard, with perioperative chemotherapy and, in selected settings, preoperative chemoradiotherapy improving outcomes compared with surgery alone [3–6]. Despite these advances, long-term outcomes remain unsatisfactory, highlighting the need for more effective preoperative treatment strategies.

Immune checkpoint inhibitors (ICIs) have substantially expanded treatment options in advanced gastroesophageal malignancies and are increasingly being explored in earlier-stage disease. Agents targeting the programmed death-1 (PD-1) and programmed death-ligand 1 (PD-L1) axis have shown clinically meaningful activity in advanced gastric and GEJ adenocarcinoma, supporting investigation in the neoadjuvant setting [7–9]. More broadly, the neoadjuvant use of immunotherapy has attracted interest across solid tumors because exposure to intact tumor antigen before resection may enhance systemic antitumor immune priming and potentially improve eradication of micrometastatic disease [10, 11]. Early studies of ICI-based neoadjuvant therapy in gastric and GEJ adenocarcinoma, most commonly in combination with chemotherapy, have reported encouraging pathological, radiological, and surgical outcomes. However, the available evidence remains heterogeneous, with variation in tumor site, treatment backbone, study design, and the use of postoperative therapy, complicating interpretation across neoadjuvant-only and broader perioperative treatment frameworks.

Several recent systematic reviews and meta-analyses have evaluated neoadjuvant immunotherapy in resectable gastric and GEJ malignancies, but these analyses have generally been limited by earlier search windows, narrower treatment definitions, or broader disease groupings that may not fully reflect outcomes attributable to the preoperative treatment phase [12–14]. As the evidence base continues to evolve, an updated synthesis focused specifically on locally advanced resectable gastric and GEJ adenocarcinoma remains clinically relevant, particularly when emphasizing endpoints that most directly reflect the effect of neoadjuvant treatment.

In this context, pathological and surgery-related outcomes are especially informative because they more directly capture the effect of preoperative therapy. Endpoints such as pathologic complete response (pCR), major pathologic response (MPR), objective response rate (ORR), and R0 resection rate provide early measures of antitumor activity and the likelihood of achieving curative-intent resection. At the same time, treatment-related toxicity and surgery-related safety remain important considerations when incorporating immunotherapy into multimodality care, particularly in patients proceeding to major upper gastrointestinal surgery. By contrast, longer-term outcomes such as overall survival (OS), progression-free survival (PFS), disease-free survival (DFS), and event-free survival (EFS) may be influenced by the broader treatment strategy, especially when postoperative therapy is administered, and therefore require cautious interpretation in studies evaluating neoadjuvant approaches.

Accordingly, this systematic review and meta-analysis aimed to evaluate the efficacy and safety of neoadjuvant immune checkpoint inhibitor–based therapy in adults with locally advanced resectable gastric and GEJ adenocarcinoma. The primary objective was to synthesize preoperative efficacy and surgery-related outcomes, specifically pCR, MPR, ORR, and R0 resection rate, across eligible studies, including perioperative studies only when these outcomes were clearly attributable to the preoperative treatment phase and could be extracted separately. The secondary objective was to summarize treatment-related safety outcomes, including grade ≥ 3 adverse events, immune-related adverse events (irAEs), and surgical complication rates, as well as to describe available longer-term oncologic outcomes, including OS, PFS, DFS, and EFS.

## Methods

This systematic review and meta-analysis was conducted in accordance with the Preferred Reporting Items for Systematic Reviews and Meta-Analyses (PRISMA) 2020 guidelines [15]. The methodology was predefined to promote transparency and reproducibility and was guided by the Cochrane Handbook for Systematic Reviews of Interventions [16].

### Eligibility criteria

Studies were eligible if they enrolled adults with histologically confirmed, locally advanced, resectable gastric and/or gastroesophageal junction (GEJ) adenocarcinoma and evaluated immune checkpoint inhibitor–based therapy administered in the neoadjuvant setting, either alone or in combination with systemic therapy. Eligible studies were required to report at least one prespecified outcome of interest, including pathologic complete response (pCR), major pathologic response (MPR), objective response rate (ORR), R0 resection rate, grade ≥ 3 adverse events, immune-related adverse events (irAEs), surgical complication rates, or longer-term oncologic outcomes, including overall survival (OS), progression-free survival (PFS), disease-free survival (DFS), or event-free survival (EFS). Perioperative studies were included only when preoperative efficacy or surgical outcomes (e.g., pCR, MPR, ORR, and R0 resection) were clearly attributable to the neoadjuvant phase and could be extracted separately. Because postoperative chemotherapy and/or adjuvant immunotherapy may influence longer-term outcomes, survival endpoints from perioperative studies were considered descriptive and were summarized narratively rather than pooled quantitatively.

Studies were excluded if they included unresectable or metastatic disease without separable data for the resectable cohort, did not involve neoadjuvant immune checkpoint inhibitor–based therapy, or lacked sufficient data for extraction. Case reports, review articles, editorials, letters, conference abstracts without adequate data, and non-human studies were also excluded. Only studies published in English were considered.

### Search strategy

A comprehensive literature search was conducted in PubMed, Embase (via Ovid), Cochrane CENTRAL, Web of Science, and Scopus to identify studies evaluating neoadjuvant or perioperative immune checkpoint inhibitor (ICI)-based therapy in patients with locally advanced resectable gastric and gastroesophageal junction (GEJ) adenocarcinoma. Google Scholar was additionally searched to identify grey literature. The search covered the period from 2014 to 2026 and was limited to human studies published in English.

The search strategy combined controlled vocabulary terms, where available, with free-text keywords using Boolean operators. Search terms were tailored to each database and were structured around three core concepts: gastric or GEJ adenocarcinoma, the neoadjuvant or perioperative treatment setting, and immune checkpoint inhibition. The reference lists of included studies and relevant review articles were also screened manually to identify any additional eligible studies. The primary search strategy was based on the following terms:

(“gastric cancer” OR “gastric adenocarcinoma” OR “stomach cancer” OR “gastroesophageal junction cancer” OR “gastroesophageal junction adenocarcinoma” OR “esophagogastric junction cancer” OR “GEJ cancer” OR “EGJ cancer”) AND (“neoadjuvant” OR “preoperative” OR “perioperative”) AND (“immunotherapy” OR “immune checkpoint inhibitor” OR “checkpoint inhibitor” OR “PD-1” OR “PD-L1” OR “CTLA-4” OR pembrolizumab OR nivolumab OR sintilimab OR camrelizumab OR tislelizumab OR toripalimab OR atezolizumab OR durvalumab OR avelumab OR ipilimumab).

The complete database-specific search strategies are provided in Supplementary Appendix Table S1.

### Study selection

Following completion of the database searches, all retrieved records were imported into EndNote X9 (Clarivate Analytics) for reference management [17], and duplicate records were removed prior to screening. The remaining studies were screened at the title and abstract level according to the predefined eligibility criteria. Screening was performed independently by two reviewers, with disagreements resolved through discussion and consensus. Full-text articles of potentially eligible studies were subsequently assessed to confirm final eligibility. Studies that met all inclusion criteria and evaluated neoadjuvant immune checkpoint inhibitor-based therapy in adults with locally advanced resectable gastric and/or gastroesophageal junction (GEJ) adenocarcinoma were included in the qualitative synthesis and, where appropriate, the quantitative meta-analysis.

### Data extraction

Data extraction was performed independently by two reviewers using a standardized data extraction form. Extracted data included study characteristics (first author, year of publication, study design, and country), cohort characteristics (sample size of the immune checkpoint inhibitor cohort and tumor site), treatment-related variables (treatment strategy and neoadjuvant immune checkpoint inhibitor–based regimen), reported outcomes (including efficacy, safety, and survival endpoints), and key study findings. Where studies evaluated perioperative treatment strategies, only data relevant to the preoperative treatment phase, particularly preoperative efficacy and surgical outcomes, were extracted where feasible. Any discrepancies between reviewers were resolved through discussion and consensus.

### Outcome measures

The primary outcomes were preoperative efficacy and surgical endpoints, including pathologic complete response (pCR), major pathologic response (MPR), objective response rate (ORR), and R0 resection rate. These outcomes were chosen to reflect short-term treatment activity and the likelihood of achieving curative-intent resection after neoadjuvant or perioperative immune checkpoint inhibitor (ICI)-based therapy.

Secondary outcomes included safety endpoints (grade ≥ 3 adverse events, immune-related adverse events [irAEs], and surgical complication rates) and longer-term oncologic outcomes (overall survival [OS], progression-free survival [PFS], disease-free survival [DFS], and event-free survival [EFS]), where reported. Because consistently extractable time-to-event estimates were unavailable, OS and DFS were summarized descriptively using reported death and recurrence events, respectively, when available, based on data from the included studies. In studies evaluating perioperative strategies, survival outcomes were interpreted cautiously because postoperative chemotherapy, adjuvant immunotherapy, or maintenance treatment may have contributed to the observed effect.

### Data synthesis and analysis

A qualitative synthesis was first performed to summarize the characteristics of included studies, including study design, patient population, treatment regimens, and reported oncologic and safety outcomes. Where studies were sufficiently comparable in design and outcome reporting, quantitative synthesis was undertaken.

For the meta-analysis, pooled proportions were calculated for single-arm outcomes, including pathologic complete response (pCR), major pathologic response (MPR), objective response rate (ORR), R0 resection, grade 3 or higher treatment-related adverse events, immune-related adverse events, and postoperative surgical complications. Random-effects models were used throughout to account for anticipated clinical and methodological heterogeneity across studies [18]. Pooled estimates are presented with corresponding 95% confidence intervals (CIs).

Statistical heterogeneity was assessed using Cochran’s Q test and quantified using the I² statistic [19]. Sensitivity analyses were performed using leave-one-out influence analysis to assess the robustness of pooled estimates. Prespecified subgroup analyses according to regimen intensity, study design, and treatment strategy were conducted to explore potential sources of heterogeneity. Meta-regression was considered but was not performed because the limited number of studies within several clinically relevant categories and the substantial overlap between study characteristics were unlikely to yield robust or reliable estimates. Publication bias was evaluated by visual inspection of funnel plots and Egger’s regression test when sufficient studies were available [20]. All statistical analyses were performed using R statistical software [21].

### Risk of bias assessment

Risk of bias was assessed according to study design using established methodological appraisal tools. Randomized controlled trials were evaluated using the Cochrane Risk of Bias 2 (RoB 2) tool [22], Non-randomized comparative cohort studies were assessed using the Newcastle–Ottawa Scale (NOS) [23], and non-comparative single-arm studies were evaluated using the 8-item non-comparative version of the Methodological Index for Non-Randomized Studies (MINORS) [24].

## Results

### Study selection outcome

**Fig. 1:**
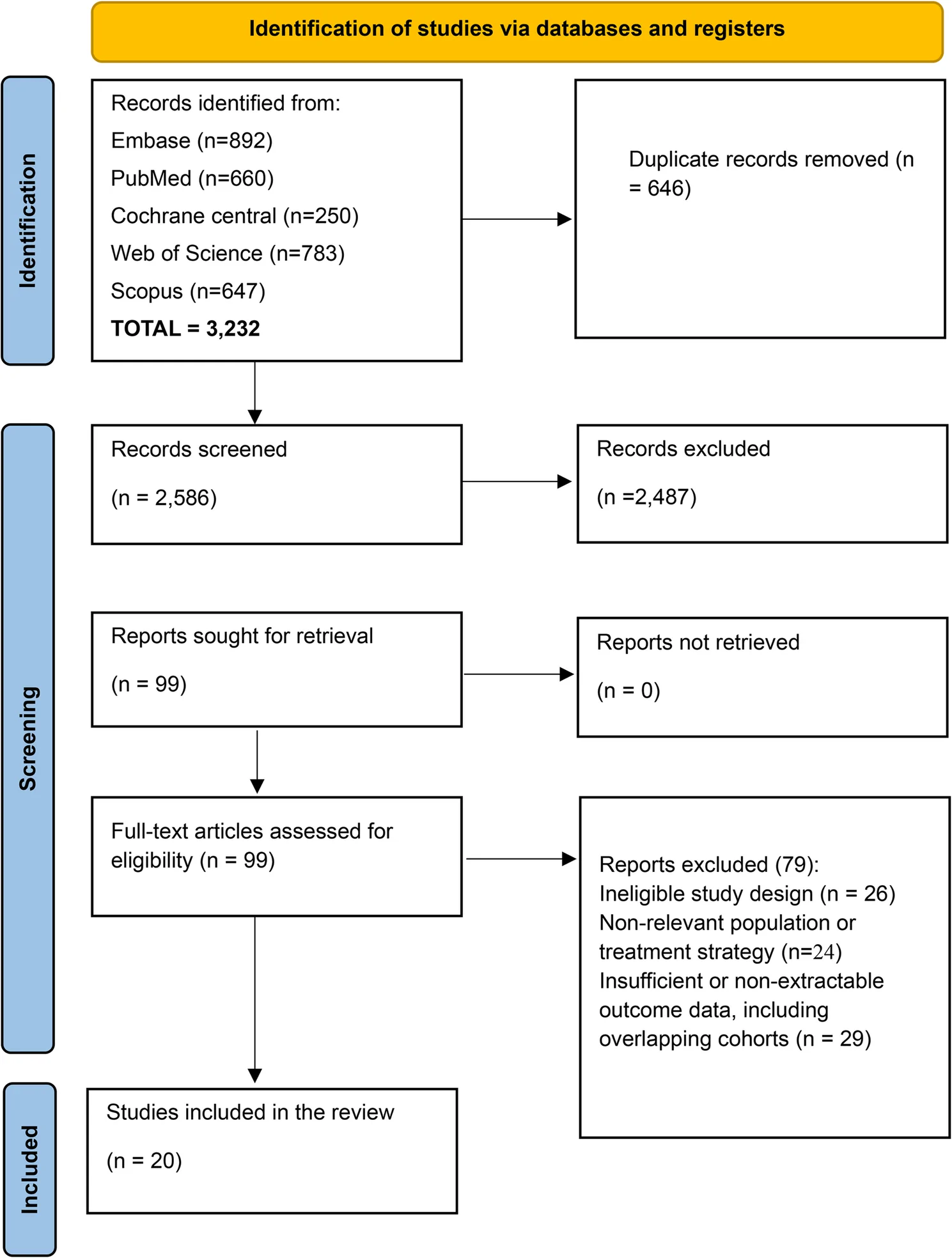
PRISMA flowchart showing the study selection process

The literature search identified 3,232 records across five electronic databases: PubMed/MEDLINE (*n* = 660), Embase via Ovid (*n* = 892), Cochrane CENTRAL (*n* = 250), Web of Science (*n* = 783), Scopus (*n* = 647). After removal of 646 duplicate records, 2,586 studies remained for title and abstract screening. Of these, 2,487 were excluded as clearly irrelevant to the review question. A total of 99 full-text articles were assessed for eligibility, of which 79 were excluded due to non-original or ineligible study design, non-relevant population or treatment strategy, or insufficient or non-extractable outcome data, including overlapping cohorts. Ultimately, 20 studies met the predefined inclusion criteria and were included in both the qualitative synthesis and the quantitative meta-analysis. The study selection process is summarised in the PRISMA flow diagram in Fig. 1.

### Quality assessment outcome

**Fig. 2:**
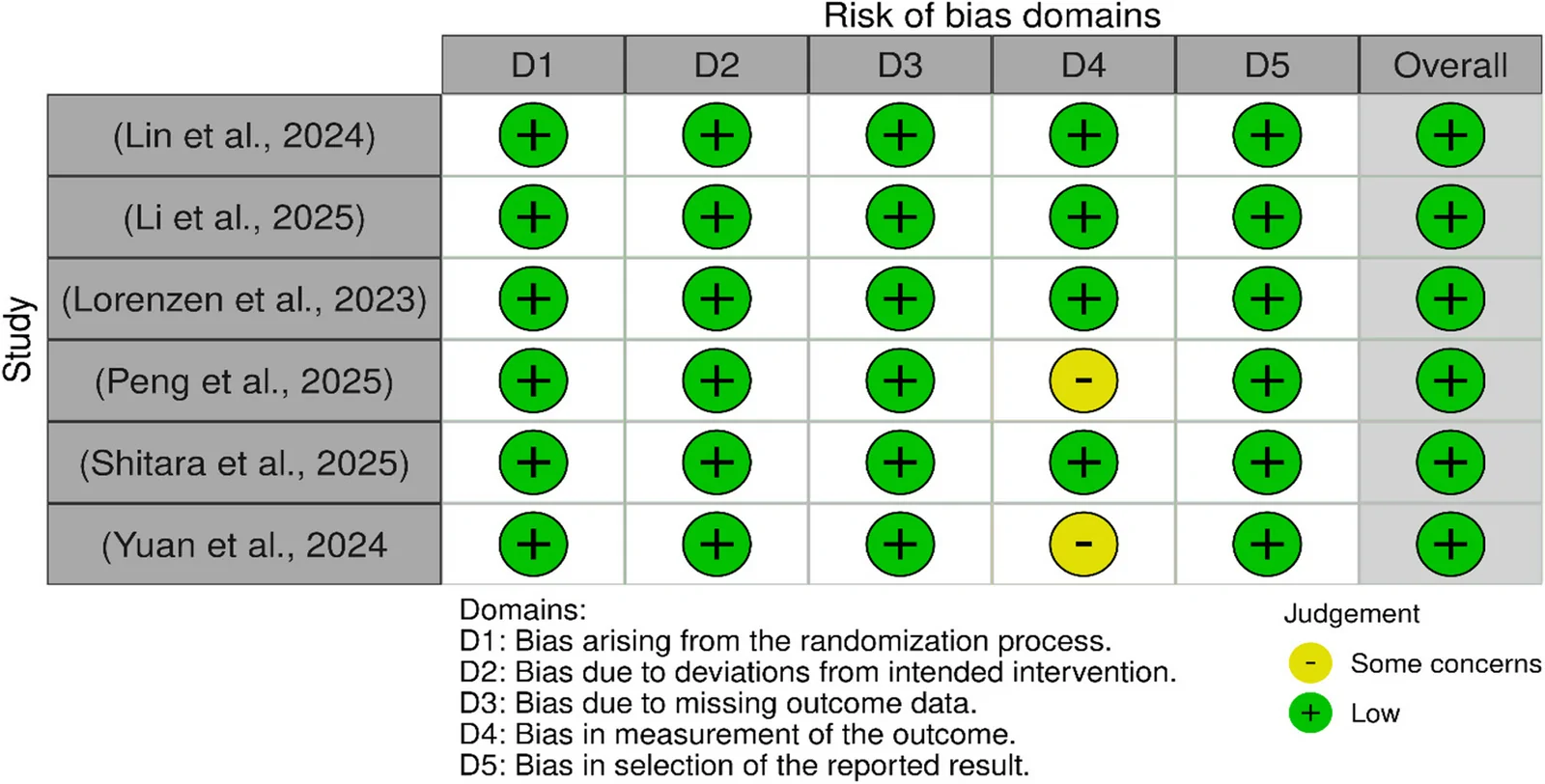
Traffic light plot of included RCTs

All six randomized trials were judged overall as low risk of bias according to RoB 2 [25–30], Although two studies had some concerns in the outcome measurement domain that did not alter the overall judgment, and both studies remained classified as low risk [28, 30] (Fig. 2).

**Fig. 3:**
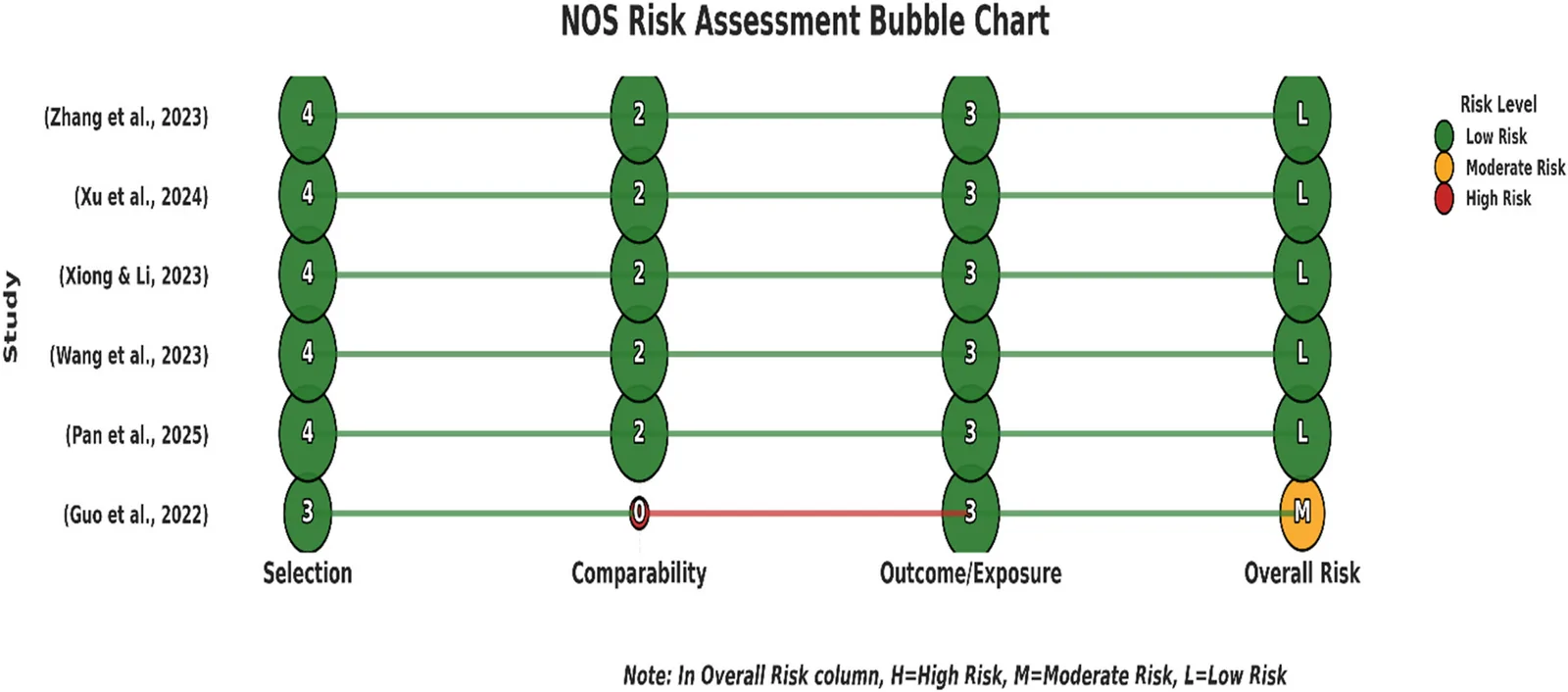
Newcastle–Ottawa Scale (NOS) for the included studies

Among the six comparative cohort studies, five were considered low risk, and one was considered moderate risk [31–36]. The moderate-risk study lost points in the comparability domain, whereas the remaining studies demonstrated strong performance in cohort selection, comparability, and outcome assessment. Overall, the comparative observational evidence was methodologically sound (Fig. 3).

The eight single-arm studies showed generally good methodological quality, with total scores ranging from 11 to 16 out of 16 [37–44]. Four studies achieved the maximum score of 16 [37, 38, 40, 41], three studies scored 15 [39, 43, 44], and one study scored 11 [42]. The lower score in Tang et al. [42] was mainly related to the absence of prospective data collection and prospective sample size calculation, together with less robust follow-up reporting. Across the single-arm studies, the domain most commonly not fully satisfied was prospective sample size calculation (Table 1).

**Table 1:** Methodological quality assessment of single-arm studies using the non-comparative MINORS instrument

Study	Clearly stated aim (0–2)	Consecutive patients (0–2)	Prospective data collection (0–2)	Endpoints appropriate (0–2)	Unbiased endpoint assessment (0–2)	Adequate follow-up (0–2)	Loss to follow-up < 5% (0–2)	Prospective sample size calculation (0–2)	Total (0–16)
Jiang et al., 2022 [38]	2	2	2	2	2	2	2	2	16
Li et al., 2024 [39]	2	2	2	2	2	2	2	1	15
Li et al., 2023 [40]	2	2	2	2	2	2	2	2	15
Sun et al., 2024 [41]	2	2	2	2	2	2	2	2	15
Tang et al., 2022 [42]	2	2	0	2	2	1	2	0	11
Verschoor et al., 2024 [43]	2	2	2	2	2	2	2	1	15
Zhong et al., 2024 [44]	2	2	2	2	2	2	2	1	15
André et al., 2023 [37]	2	2	2	2	2	2	2	2	16

Overall. the included studies were of acceptable to high methodological quality, with uniformly low risk across the randomized evidence, predominantly low risk among comparative observational studies, and generally favorable quality among the single-arm cohorts

### Characteristics of Included Studies

The 20 included studies comprised six randomized controlled trials (RCTs), six comparative observational cohort studies, and eight single-arm studies evaluating neoadjuvant or perioperative immune checkpoint inhibitor (ICI)-based strategies in resectable locally advanced gastric and gastroesophageal junction (GEJ) adenocarcinoma. Most studies were conducted in China, with additional studies from the Netherlands, France, and Germany/Switzerland, as well as one multinational randomized trial that enrolled patients across Asia, Europe, and the Americas [27, 29, 37, 43].

Sample sizes varied substantially across study designs. Among the RCTs, ICI-treated cohorts ranged from 21 to 402 patients, with Shitara et al. [29] representing the largest study and Li et al. [25] and Lorenzen et al. [27] also contributing relatively large randomized datasets. Comparative cohort studies included 30–201 patients in the ICI-treated cohorts, with the largest real-world matched cohort reported by Xu et al. [35]. Single-arm studies were generally smaller, typically enrolling 21 to 75 patients, although some reported fewer patients evaluable for pathologic response or efficacy [35, 38, 42, 43].

All studies enrolled patients treated with curative intent for resectable, locally advanced gastric adenocarcinoma and/or GEJ adenocarcinoma, although the relative proportion of gastric and GEJ tumors varied considerably across cohorts. Several studies included mixed gastric and GEJ populations, whereas some cohorts were predominantly gastric cancer, particularly in the Chinese studies [25, 26, 31]. By contrast, GEJ tumors represented a larger proportion in some European cohorts, including Verschoor et al. [43] and Lorenzen et al. [27]. One prospective single-arm study also included a conversion-therapy population with cT4a/bN+ locally advanced disease, reflecting a somewhat more advanced clinical subgroup than the predominantly resectable cohorts included elsewhere [40].

Treatment strategies were heterogeneous but consistently centered on the incorporation of ICIs into neoadjuvant or perioperative systemic therapy. Several studies evaluated pure neoadjuvant immunochemotherapy followed by surgery, whereas others used perioperative protocols that incorporated postoperative chemotherapy and, in selected trials, continued adjuvant or maintenance immunotherapy [25, 27, 29–31, 39, 41]. Most regimens were built on fluoropyrimidine-platinum chemotherapy backbones, including SOX, XELOX/CapeOx, FOLFOX, and FLOT. The most frequently evaluated ICIs were camrelizumab, sintilimab, tislelizumab, toripalimab, pembrolizumab, atezolizumab, and nivolumab. Some studies incorporated targeted therapy, particularly VEGFR inhibition with apatinib or rivoceranib, while one HER2-directed perioperative RCT combined atezolizumab with trastuzumab and XELOX. In addition, one French prospective study evaluated dual checkpoint blockade with nivolumab plus ipilimumab in a chemotherapy-free perioperative strategy [25, 28, 33, 34, 37, 40].

The reported study endpoints were also diverse but showed clear thematic consistency across the literature. Pathologic response outcomes, particularly pathologic complete response (pCR) and major pathologic response (MPR), were the most frequently reported efficacy endpoints. R0 resection rate was reported in nearly all studies and served as a key surgical feasibility outcome. Additional short-term efficacy endpoints variably included objective response rate (ORR), disease control rate (DCR), pathologic partial response (PPR), total pathologic complete response (tpCR/ypT0N0), tumor regression grading (TRG), pathologic response, and TNM downstaging. Longer-term oncologic outcomes were less consistently mature but included disease-free survival (DFS), progression-free survival (PFS), event-free survival (EFS), recurrence-free survival (RFS), and overall survival (OS). Safety reporting was generally comprehensive and included grade ≥ 3 treatment-related adverse events (TRAEs), immune-related adverse events (irAEs), and perioperative surgical morbidity or complication rates, with some RCTs additionally reporting surgical mortality and patient-reported outcomes [26, 27, 29–31, 39, 44].

Overall, the included studies evaluated clinically diverse treatment approaches rather than a single uniform intervention. Regimens ranged from ICI plus chemotherapy, ICI combined with antiangiogenic therapy, ICI combined with HER2-directed therapy, chemotherapy-free dual immune checkpoint blockade, and perioperative strategies incorporating variable postoperative treatments. These differences likely represent biologically distinct therapeutic paradigms with potentially different mechanisms of response and toxicity. Consequently, pooled estimates should be interpreted as reflecting the overall activity of ICI-containing preoperative strategies rather than the effect of any specific regimen. Furthermore, postoperative treatment varied considerably across perioperative studies, introducing an additional source of heterogeneity when comparing long-term outcomes. This is shown in Table 2 in the Appendix.

**Table 2:** Characteristics of Included Studies

Study, Design	Country	Sample size (ICI cohort)	Tumor Site	Treatment Strategy	Neoadjuvant ICI-based Regimen	Outcomes Reported	Key findings
(Guo et al., 2022) [31], Retrospective, comparative cohort study.	China	30 patients	Gastric adenocarcinoma (locally advanced, resectable)	Perioperative (4 cycles neoadjuvant + surgery + 4 cycles adjuvant)	Sintilimab (anti–PD-1) + XELOX (oxaliplatin + capecitabine)	pCR, MPR, ORR, DCR, R0 resection rate, TNM downstaging, DFS, OS, surgical complications, irAEs, Grade ≥ 3 TRAEs	High pCR (33.3%) and MPR (63.3%) with 100% R0 resection; manageable toxicity and acceptable surgical safety
(Jiang et al., 2022) [38], Prospective single-arm	China	36 patients	Gastric adenocarcinoma (80.6%) and GEJ adenocarcinoma (19.4%)	Neoadjuvant	Sintilimab (anti–PD-1) + CapeOx (oxaliplatin + capecitabine)	pCR, MPR, ORR, TNM downstaging, R0 resection rate, DFS, OS, surgical complications, irAEs, Grade ≥ 3 TRAEs	pCR 19.4%, MPR 47.2%, R0 resection 97.2%; encouraging efficacy with manageable toxicity and high 1-year DFS/OS rates
(Li et al., 2024) [39], single-arm prospective cohort study	China	32 patients	Gastric adenocarcinoma (81.3%) and GEJ adenocarcinoma (18.7%)	Perioperative (3 cycles sintilimab + 4 cycles FLOT pre-op, surgery, then 4 cycles adjuvant FLOT)	Sintilimab (anti–PD-1) + FLOT (fluorouracil, leucovorin, oxaliplatin, docetaxel)	pCR, MPR, ORR, DCR, R0 resection rate, TNM downstaging, DFS, OS, EFS, surgical complications, irAEs, Grade ≥ 3 TRAEs	pCR 17.2%, MPR 55.2%, R0 resection 93.1%; 3-year DFS 78.8%, OS 70.9%; manageable toxicity, no grade ≥ 3 surgical complications
(Li et al., 2023) [40], Prospective, single-arm study	China	25 patients enrolled; 19 evaluable for pathological response	Gastric adenocarcinoma (locally advanced, cT4a/bN+)	Neoadjuvant/conversion	Camrelizumab (anti–PD-1) + apatinib (VEGFR2 TKI) + S-1 (± oxaliplatin)	pCR, MPR, PPR, ORR, tumor downstaging, R0 resection rate, DFS, OS, surgical complications, irAEs, Grade ≥ 3 TRAEs	pCR 15.8%, MPR 26.3%, R0 resection 82.6%; high downstaging rate (76%) with limited toxicity and acceptable surgical safety
(Lin et al., 2024) [26], Multicenter, RCT	China	51 patients	Gastric adenocarcinoma (clinical T2–4 N + M0)	Neoadjuvant	Camrelizumab (anti–PD-1) + apatinib (VEGFR2 TKI) + nab-paclitaxel + S-1	pCR, MPR, ORR, DCR, R0 resection rate, TNM downstaging, surgical complications, irAEs, Grade ≥ 3 TRAEs	MPR 33.3%, pCR 15.7%, ORR 66%, R0 resection 94.1%; toxicity manageable, surgical safety acceptable
(Pan et al., 2025) [32], Single-center, retrospective real-world study	China	49 patients received PD-1 inhibitor + chemotherapy	Gastric adenocarcinoma (95.5%) and GEJ adenocarcinoma (small proportion)	Neoadjuvant	PD-1 inhibitors (sintilimab, tislelizumab, nivolumab, toripalimab, pembrolizumab) + chemotherapy (SOX, CapeOx, nab-paclitaxel + S-1)	pCR, MPR, TNM downstaging, R0 resection rate, DFS, OS, surgical complications, irAEs, Grade ≥ 3 TRAEs	pCR 22.4% vs. 4.8% (chemo alone), 100% R0 resection; toxicity manageable, no excess surgical risk
(Sun et al., 2024) [41], Prospective, single-arm	China	49 patients	Gastric adenocarcinoma (75.5%) and GEJ adenocarcinoma (24.5%)	Perioperative (3 cycles neoadjuvant + surgery + 5 cycles adjuvant SOX)	Tislelizumab (anti–PD-1) + SOX (S-1 + oxaliplatin)	pCR, MPR, R0 resection rate, TNM downstaging, PFS, OS, surgical complications, irAEs, Grade ≥ 3 TRAEs	pCR 26.5%, MPR 49.0%, R0 resection 100%; 2-year PFS 69.4%, OS 81.2%; toxicity acceptable, one fatal irAE (HLH)
(Tang et al., 2022) [42], Retrospective, single-arm observational study	China	75 patients	Gastric adenocarcinoma (72.5%) and GEJ adenocarcinoma (27.5%	Neoadjuvant	Camrelizumab (anti–PD-1) + FOLFOX (oxaliplatin + leucovorin + 5-FU)	pCR, MPR, ORR, R0 resection rate, TNM downstaging, DFS, OS, surgical complications, irAEs, Grade ≥ 3 TRAEs	pCR 20%, MPR 45%, R0 resection 95%; encouraging efficacy with acceptable toxicity and surgical safety
(Verschoor et al., 2024) [43], Prospective, single-arm	Netherlands	21 patients enrolled; 20 evaluable for efficacy	Gastric adenocarcinoma (24%) and GEJ adenocarcinoma (76%)	Pure neoadjuvant	Atezolizumab (anti–PD-L1) + docetaxel + oxaliplatin + capecitabine	pCR, MPR, R0 resection rate, DFS, OS, surgical complications, irAEs, Grade ≥ 3 TRAEs	MPR 70%, pCR 45%, R0 resection 95%; excellent correlation between pathologic response and survival, manageable toxicity
(Wang et al., 2023) [33], Prospective, non-randomized cohort study	China	39 patients	Gastric adenocarcinoma (76.9%) and GEJ adenocarcinoma (23.1%)	Neoadjuvant	PD-1 inhibitors (sintilimab, camrelizumab, or toripalimab) + apatinib + SOX/CapeO	pCR, pathologic response, ORR, DCR, R0 resection rate, DFS, PFS, OS, surgical complications, irAEs, Grade ≥ 3 TRAEs	PAC group showed higher ORR (74.4%), pCR (23.1%), R0 resection (97.4%), and prolonged 3-year PFS (76.1%) and OS (86.7%) compared to AC; toxicity manageable, no excess surgical risk
(Xiong & Li, 2023) [34], Retrospective, comparative cohort study	China	56 patients	Gastric adenocarcinoma (76.8%) and GEJ adenocarcinoma (23.2%)	Neoadjuvant	Sintilimab or camrelizumab (PD-1 inhibitor) + apatinib + SOX/CapeOx	ORR, DCR, pCR, pathological response, R0 resection rate, PFS, DFS, OS, adverse events	PAC group achieved higher ORR (73.2%), pCR (26.8%), and longer PFS/OS vs. chemo alone; toxicity acceptable, peripheral neuropathy slightly increased
(Xu et al., 2024) [35], Multicenter, retrospective, real-world cohort study	China	201 patients received ICIs + chemotherapy (184 matched for analysis)	Gastric adenocarcinoma and GEJ adenocarcinoma	Neoadjuvant	Anti-PD-1/PD-L1 ICIs (various agents) + doublet/triplet chemotherapy (SOX, XELOX, FLOT, DOS, DCF, DCX)	pCR, ORR, DFS, OS, R0 resection rate, surgical complication rate	ICIs + chemo improved pCR (14.1% vs. 7.6%) and 2–3 year DFS, with no excess surgical complications; OS benefit not significant
(Zhang et al., 2023) [36], Retrospective, single-center comparative cohort study	China	34 patients	Gastric adenocarcinoma (76.5%) and GEJ adenocarcinoma (23.5%)	Neoadjuvant	Camrelizumab, sintilimab, nivolumab, or toripalimab + FLOT or SOX	pCR, MPR, ORR, R0 resection rate, TNM downstaging, RFS, OS, surgical complications, Grade ≥ 3 TRAEs	pCR 23.5% vs. 4.7% (chemo), MPR 38.2%, R0 resection 97.1%; improved pCR with acceptable toxicity
(Zhong et al., 2024) [44], Prospective, single-arm study	China	29 patients	Gastric adenocarcinoma (41.4%) and GEJ adenocarcinoma (58.6%)	Perioperative (3 cycles neoadjuvant + surgery + 3 cycles adjuvant)	Camrelizumab (anti–PD-1) + oxaliplatin + S-1 (SOX regimen)	pCR, tpCR, MPR, ORR, DCR, R0 resection rate, TNM downstaging, EFS, OS, surgical complications, irAEs, Grade ≥ 3 TRAEs	pCR 10.3%, MPR 69%, R0 resection 96.6%; modest pCR but high MPR with excellent safety (no grade ≥ 3 AEs)
(André et al., 2023) [37], Prospective, single-arm study	France	32 patients	Gastric adenocarcinoma (50%) and GEJ adenocarcinoma (50%)	Perioperative (12 weeks neoadjuvant nivolumab + ipilimumab → surgery → adjuvant nivolumab for 9 cycles)	Nivolumab (anti–PD-1) + ipilimumab (anti–CTLA-4)	pCR, R0 resection rate, EFS, OS, surgical complication rate, Grade ≥ 3 TRAEs	pCR 59%, R0 resection 100%; high response rates with manageable toxicity, though surgical morbidity was notable
(Li et al., 2025) [25], Multicenter, RCT	China	180 patients	Gastric adenocarcinoma (69%) and GEJ adenocarcinoma (31%)	Perioperative (3 cycles neoadjuvant → surgery → 3 cycles adjuvant + extended camrelizumab/rivoceranib maintenance)	Camrelizumab (anti–PD-1) + low-dose rivoceranib (VEGFR2 TKI) + SOX (S-1 + oxaliplatin)	pCR, MPR, total pCR (ypT0N0), R0 resection rate, surgical complication rate, irAEs, Grade ≥ 3 TRAEs, EFS (ongoing)	pCR 18.3% vs. 5.0% (SOX), MPR 51.1% vs. 37.8%, R0 resection 99% vs. 94%; improved pathologic response with manageable toxicity
(Lorenzen et al., 2023) [27], Multicenter, RCT	Germany & Switzerland	146 patients	Gastric adenocarcinoma (38%) and GEJ adenocarcinoma (62%)	Perioperative (4 cycles neoadjuvant + surgery + 4 cycles adjuvant + atezolizumab maintenance)	Atezolizumab (anti–PD-L1) + FLOT (fluorouracil, leucovorin, oxaliplatin, docetaxel)	pCR, MPR (TRG1a/b), R0 resection rate, ypT0, ypN0, surgical morbidity/mortality, Grade ≥ 3 TRAEs	pCR 24% vs. 15% (FLOT), MPR 49% vs. 38%, R0 resection ~ 96%; improved downstaging with similar safety profile
(Peng et al., 2025) [28], RCT	China	21 patients	Gastric adenocarcinoma (62%) and GEJ adenocarcinoma (21%)	Perioperative (3 cycles neoadjuvant + surgery + 5 cycles adjuvant)	Atezolizumab (anti–PD-L1) + trastuzumab (HER2-targeted) + XELOX (capecitabine + oxaliplatin)	pCR, MPR, ORR, R0 resection rate, EFS, DFS, OS, Grade ≥ 3 TRAEs, surgical complications	pCR 38.1% vs. 14.3% (trastuzumab + XELOX); MPR 42.9% vs. 19%; R0 resection ~ 95%; toxicity manageable, no new safety signals
(Shitara et al., 2025) [29], RCT	Multinational (Asia, Europe, Americas)	402 patients	Gastric adenocarcinoma (~ 79%) and GEJ adenocarcinoma (~ 21%)	Perioperative (3 cycles neoadjuvant + surgery + 3 cycles adjuvant chemo + 11 cycles adjuvant pembrolizumab)	Pembrolizumab (anti–PD-1) + cisplatin/capecitabine (XP) or cisplatin/5-FU (FP); separate FLOT cohort with pembrolizumab + FLOT	pCR, EFS, OS, R0 resection rate, surgical complications, Grade ≥ 3 TRAEs, patient-reported outcomes	pCR 13.4% vs. 2.0% (main cohort), EFS HR 0.81, OS HR 0.86; improved pCR and EFS trend, OS not statistically significant, safety manageable
(Yuan et al., 2024) [30], RCT	China	54 patients	Gastric adenocarcinoma (≈ 66%) and GEJ adenocarcinoma (≈ 34%)	Perioperative (3 cycles neoadjuvant + surgery + 5 cycles adjuvant + toripalimab maintenance up to 6 months)	Toripalimab (anti–PD-1) + SOX (S-1 + oxaliplatin) or XELOX (capecitabine + oxaliplatin)	pCR, MPR (TRG0/1), R0 resection rate, surgical morbidity/mortality, Grade ≥ 3 TRAEs, irAEs	TRG0/1 44.4% vs. 20.4%, pCR 22.2% vs. 7.4%; improved pathological response with manageable toxicity and no excess surgical risk

### Pathologic complete response (pCR)

Pathologic complete response (pCR) was reported in 20 studies [25–44], comprising a total of 1,532 patients who received neoadjuvant immune checkpoint inhibitor-based therapy for locally advanced resectable gastric or gastroesophageal junction adenocarcinoma. Across individual studies, the observed pCR rate varied considerably, ranging from approximately 10% to nearly 60%, reflecting differences in study design, treatment regimens, and patient populations.

**Fig. 4:**
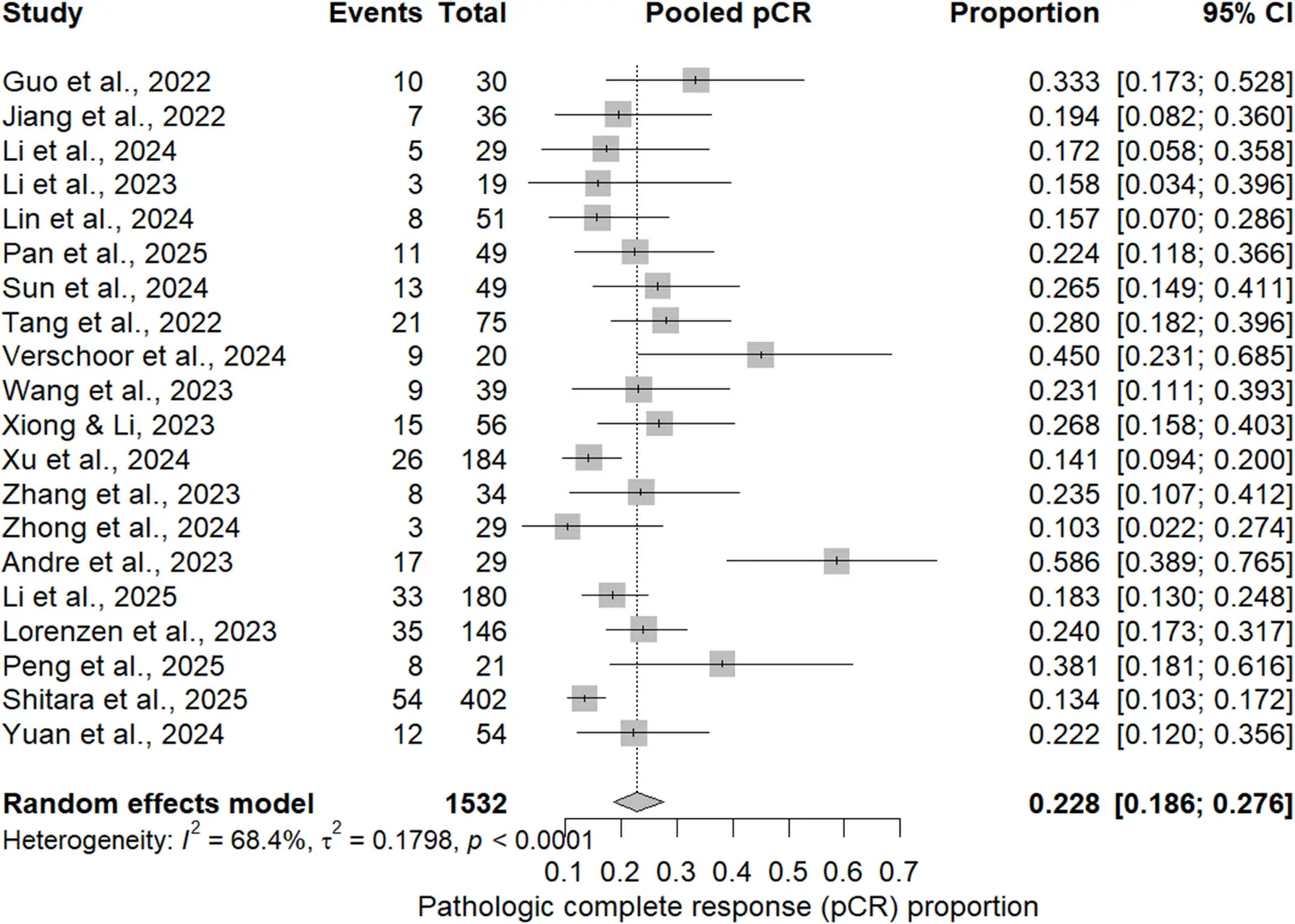
Forest plot showing the pooled pathologic complete response (pCR) rate among patients with locally advanced resectable gastric or gastroesophageal junction adenocarcinoma treated with neoadjuvant immune checkpoint inhibitor-based therapy

In the pooled random-effects analysis, the overall pCR rate was 22.8% (95% CI, 18.6%–27.6%), indicating that nearly one in four patients achieved a complete pathologic response following neoadjuvant immune checkpoint inhibitor-based treatment. Between-study heterogeneity was moderate to substantial (I² = 68.4%, *p* < 0.0001), suggesting meaningful variation in effect estimates across the included studies. Despite this heterogeneity, the pooled estimate remained consistently within a clinically relevant range and supports the activity of neoadjuvant immune checkpoint inhibitor-based strategies in this setting (Fig. 4).

### Major pathologic response (MPR)

Major pathologic response (MPR) was reported in 19 studies [25–28, 30–44], comprising a total of 1,130 patients treated with neoadjuvant immune checkpoint inhibitor-based therapy for locally advanced resectable gastric or gastroesophageal junction adenocarcinoma. Across the individual studies, MPR rates varied widely, ranging from approximately 20% to nearly 77%, reflecting notable differences in treatment regimens, study populations, and study design.

**Fig. 5:**
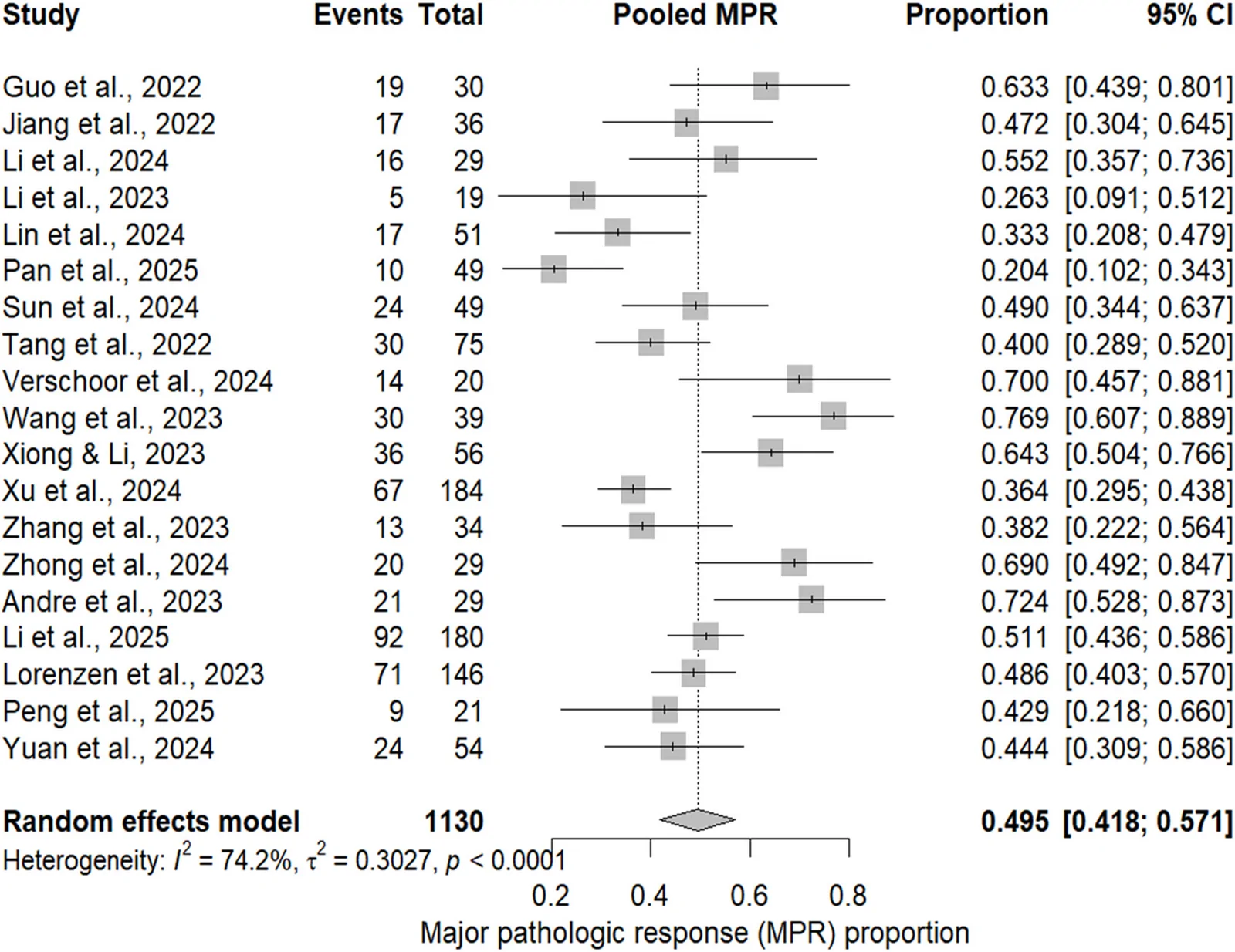
Forest plot showing the pooled major pathologic response (MPR) rate among patients with locally advanced resectable gastric or gastroesophageal junction adenocarcinoma treated with neoadjuvant immune checkpoint inhibitor-based therapy

In the pooled random-effects analysis, the overall MPR rate was 49.5% (95% CI, 41.8%–57.1%), indicating that nearly half of patients achieved a major pathologic response after neoadjuvant immune checkpoint inhibitor-based treatment. Between-study heterogeneity was substantial (I² = 74.2%, *p* < 0.0001), consistent with the broad range of MPR estimates observed across the included studies. Despite this heterogeneity, the pooled estimate remained within a clinically meaningful range across studies (Fig. 5).

### Objective response rate (ORR)

Objective response rate was reported in 12 studies [26, 28, 31, 33–40, 44] including 569 patients treated with neoadjuvant immune checkpoint inhibitor-based therapy for locally advanced resectable gastric or gastroesophageal junction adenocarcinoma. Reported ORR varied across studies, ranging from 11.1% to 93.1%, indicating substantial variability in radiologic response among the included cohorts.

**Fig. 6:**
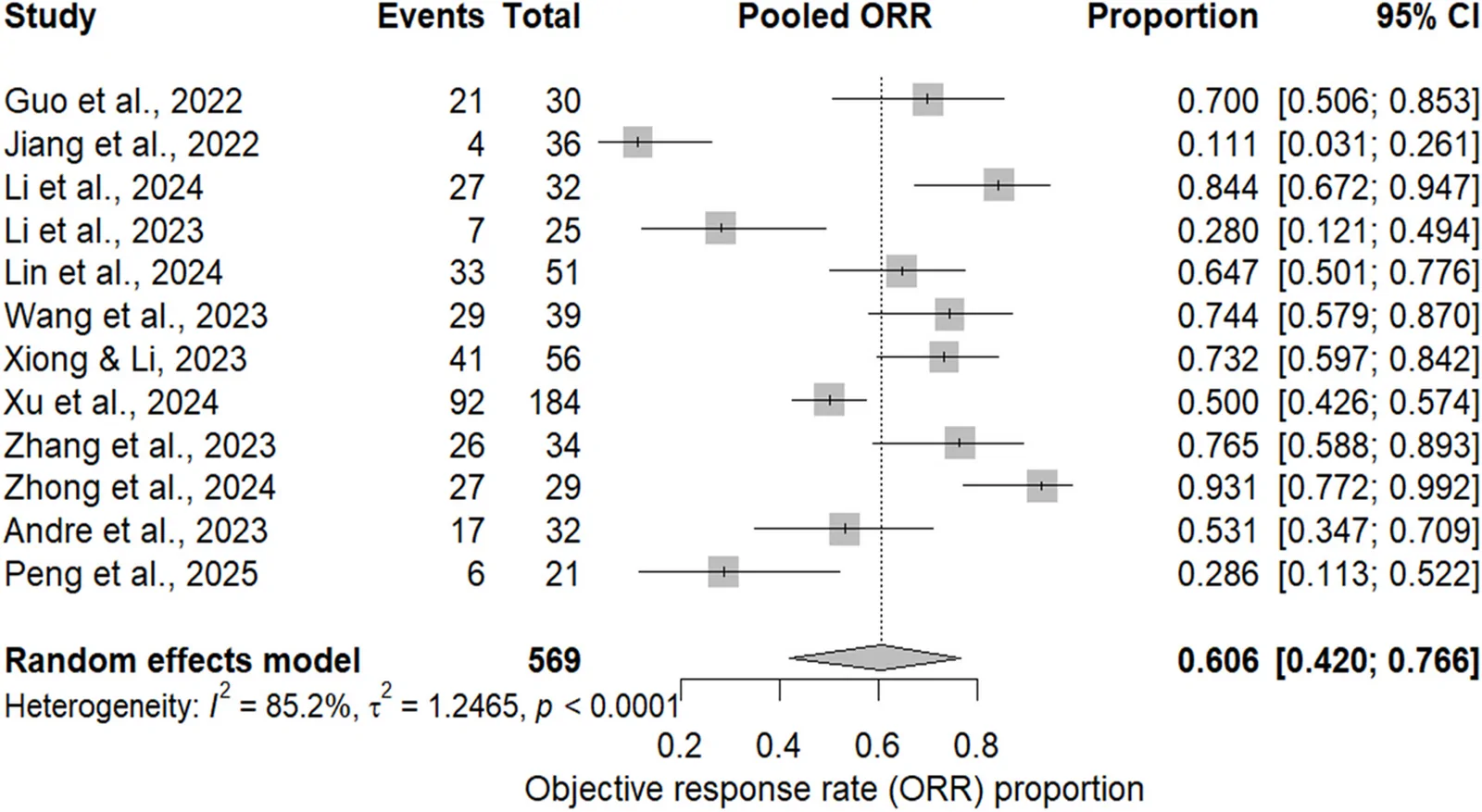
Forest plot showing the pooled objective response rate among patients with locally advanced resectable gastric or gastroesophageal junction adenocarcinoma treated with neoadjuvant immune checkpoint inhibitor-based therapy

In the random-effects model, the pooled ORR was 60.6% (95% CI, 42.0%–76.6%). Between-study heterogeneity was substantial (I² = 85.2%, *p* < 0.0001). Despite this heterogeneity, the pooled estimate remained above 50% across the included studies (Fig. 6).

### R0 resection rate

R0 resection rate was reported in 19 studies [25–28, 30–44] comprising a total of 1,102 patients treated with neoadjuvant immune checkpoint inhibitor-based therapy for locally advanced resectable gastric or gastroesophageal junction adenocarcinoma. Across the included studies, the observed R0 resection rate was generally high, with most studies reporting rates above 90%, although one study reported a notably lower estimate, contributing to the overall spread of effect sizes.

**Fig. 7:**
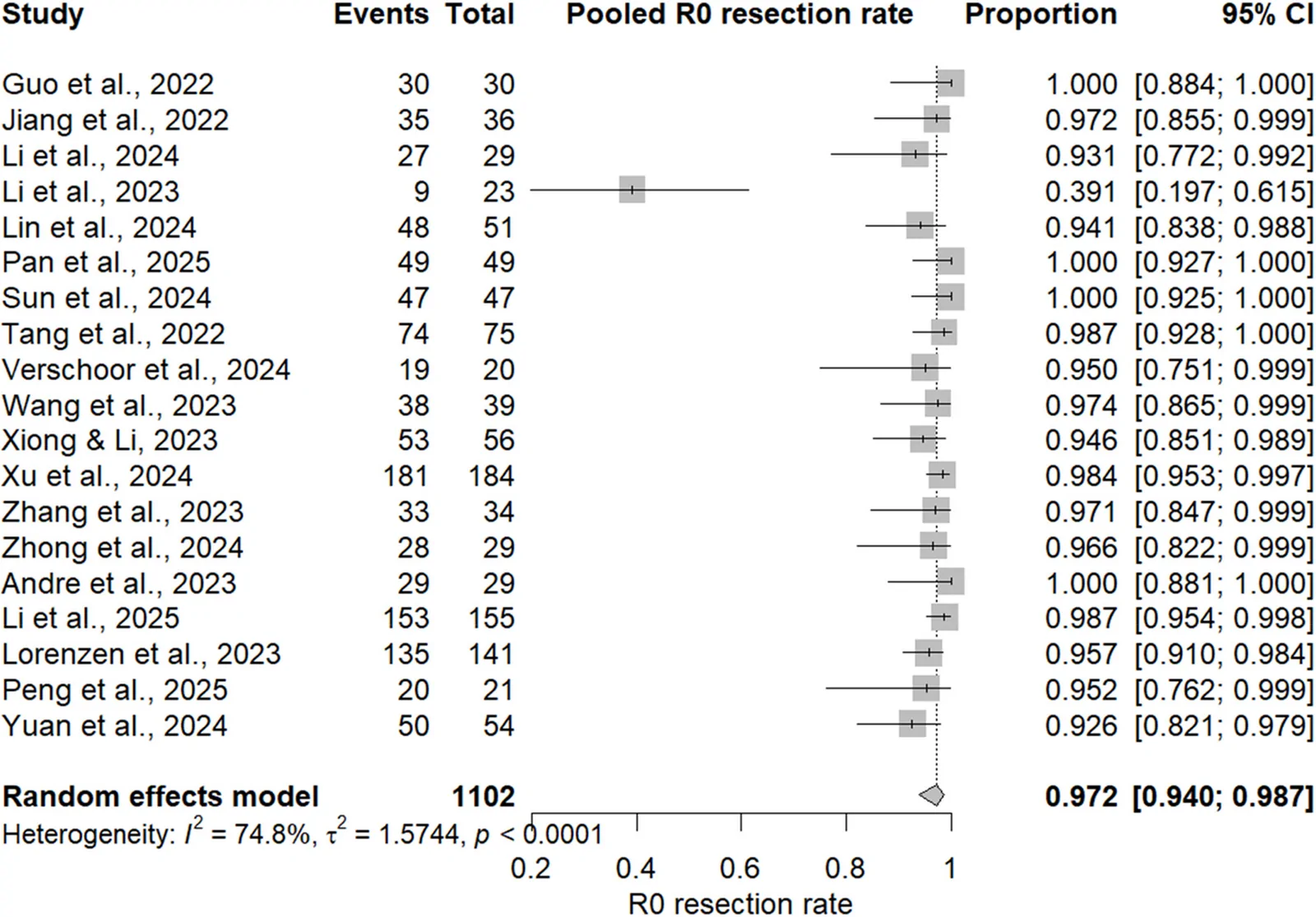
Forest plot showing the pooled R0 resection rate among patients with locally advanced resectable gastric or gastroesophageal junction adenocarcinoma treated with neoadjuvant immune checkpoint inhibitor-based therapy

In the pooled random-effects analysis, the overall R0 resection rate was 97.2% (95% CI, 94.0%–98.7%), indicating that complete microscopically margin-negative resection was achieved in the vast majority of patients undergoing surgery after neoadjuvant immune checkpoint inhibitor-based treatment. Between-study heterogeneity was substantial (I² = 74.8%, *p* < 0.0001), reflecting variability across studies despite the consistently high rates observed in most cohorts. Despite the observed heterogeneity, the pooled estimate remained consistently high across studies (Fig. 7).

### Grade ≥ 3 adverse events

Grade ≥ 3 treatment-related adverse events were reported in 17 studies [25, 26, 28, 29, 31–41, 43, 44] comprising a total of 1,135 patients treated with neoadjuvant immune checkpoint inhibitor-based therapy for locally advanced resectable gastric or gastroesophageal junction adenocarcinoma. The incidence of grade ≥ 3 adverse events varied substantially across studies, ranging from no reported events in one study to rates exceeding 60% in others, indicating marked variability in treatment-related toxicity across cohorts.

**Fig. 8:**
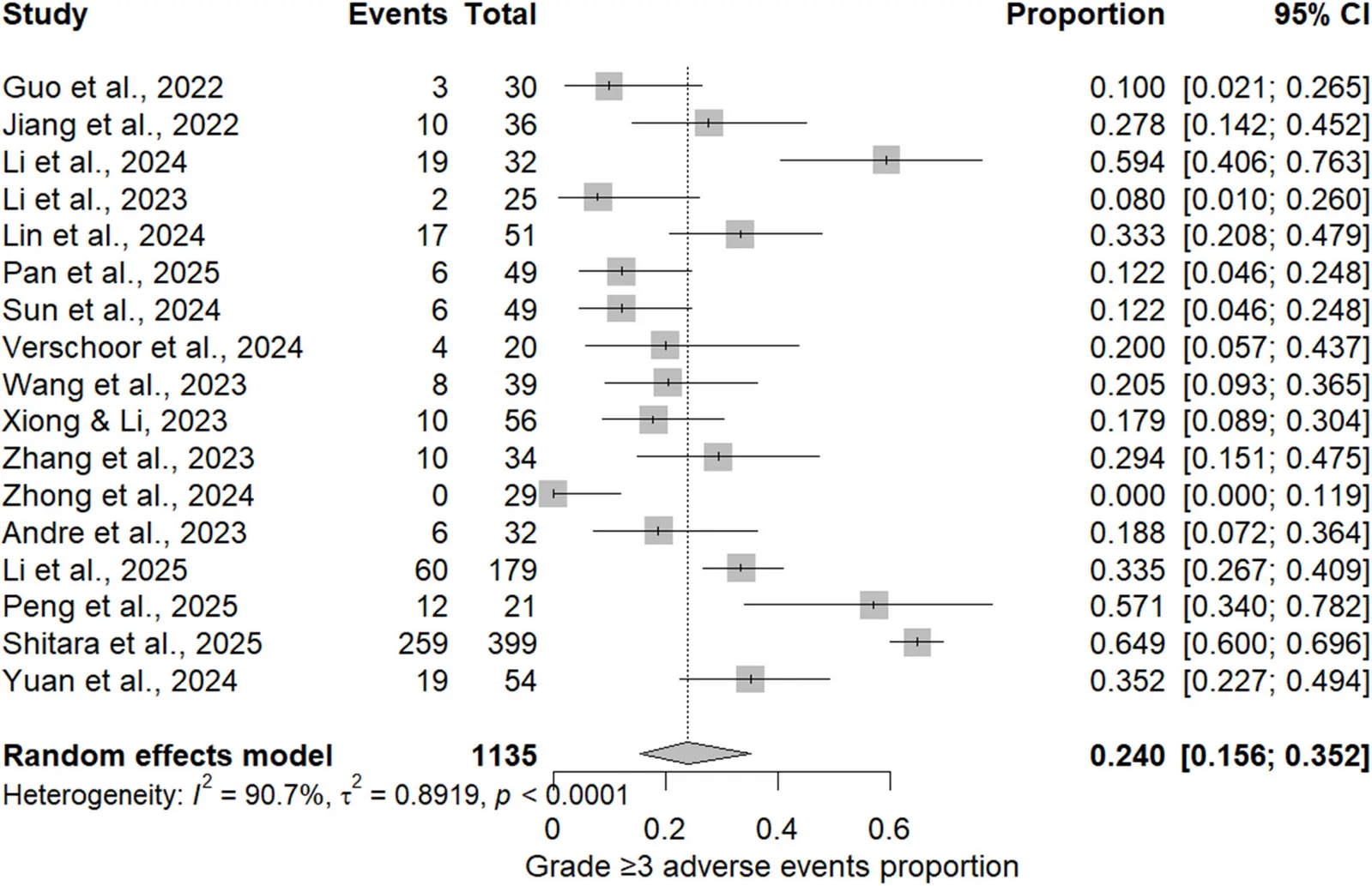
Forest plot showing the pooled proportion of grade ≥ 3 adverse events among patients with locally advanced resectable gastric or gastroesophageal junction adenocarcinoma treated with neoadjuvant immune checkpoint inhibitor-based therapy

In the pooled random-effects analysis, the overall proportion of grade ≥ 3 adverse events was 24.0% (95% CI, 15.6%–35.2%). Between-study heterogeneity was considerable (I² = 90.7%, *p* < 0.0001), reflecting substantial differences in toxicity reporting and event rates across the included studies. Despite the substantial heterogeneity, the pooled estimate indicated that grade ≥ 3 adverse events were observed in a minority of patients across the included studies (Fig. 8).

### Immune-related adverse events (irAEs).

Immune-related adverse events were reported in 14 studies [25, 26, 29–32, 36–41, 43, 44], including 1,019 patients treated with neoadjuvant immune checkpoint inhibitor-based therapy for locally advanced resectable gastric or gastroesophageal junction adenocarcinoma. Reported rates of irAEs ranged from 8.3% to 58.8%.

**Fig. 9:**
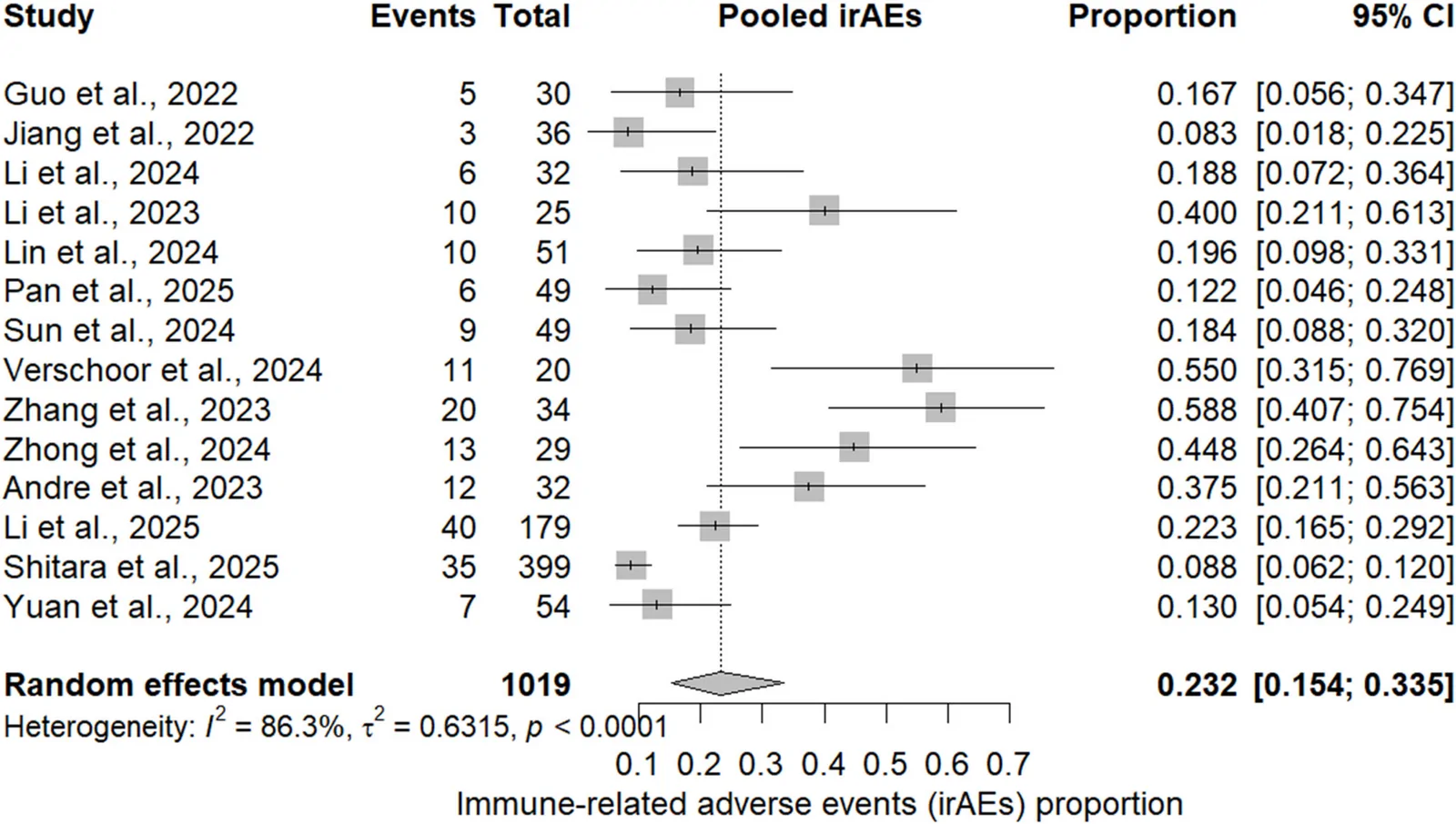
Forest plot showing the pooled proportion of immune-related adverse events among patients with locally advanced resectable gastric or gastroesophageal junction adenocarcinoma treated with neoadjuvant immune checkpoint inhibitor-based therapy

In the random-effects model, the pooled proportion of irAEs was 23.2% (95% CI, 15.4%–33.5%). Between-study heterogeneity was substantial (I² = 86.3%, *p* < 0.0001). Despite this heterogeneity, irAEs were reported in a minority of patients across the included studies (Fig. 9).

### Surgical complication rate

Surgical complication rate was reported in 15 studies [25–27, 29–32, 35, 37–40, 42–44], including 1,236 patients. Across individual studies, the proportion of patients experiencing postoperative surgical complications ranged from 9.8% to 55.2%. Pooled analysis using a random-effects model demonstrated an overall surgical complication rate of 26.2% (95% CI, 19.1%–34.7%), indicating that approximately one in four patients experienced a postoperative complication following neoadjuvant immunotherapy-based treatment and subsequent surgery.

**Fig. 10:**
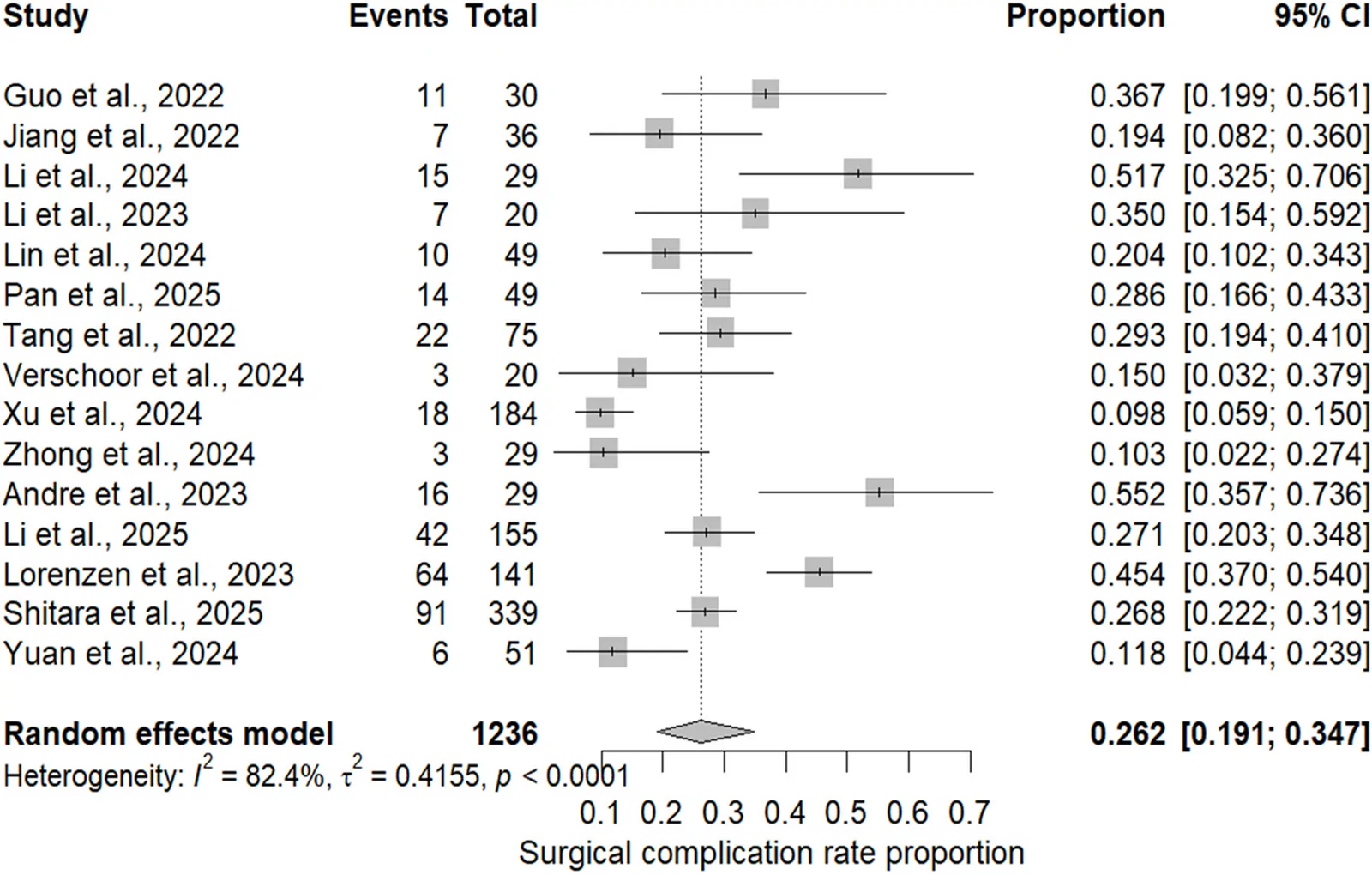
Forest plot showing the pooled proportion of postoperative surgical complications among patients undergoing surgery after neoadjuvant immunotherapy-based treatment for resectable gastric and gastroesophageal junction adenocarcinoma

There was substantial between-study heterogeneity (I² = 82.4%, *p* < 0.0001), suggesting notable variability in complication rates across included cohorts. This heterogeneity likely reflects differences in surgical techniques, perioperative management, treatment regimens, patient selection, and definitions or grading of postoperative complications across studies. Nevertheless, the pooled estimate supports the feasibility of surgery after neoadjuvant immunotherapy, with complication rates that remain within an acceptable range in the context of multimodal treatment for resectable gastric and gastroesophageal junction adenocarcinoma (Fig. 10).

### Overall survival (OS)

Overall survival outcomes were reported inconsistently across the included studies, with substantial variability in follow-up duration, sample size, and maturity of survival data. Crude event counts are presented only descriptively and should not be interpreted as comparative estimates of survival. Ten studies reported death events during follow-up, with the number of deaths ranging from 0 to 181 across cohorts [29, 31–33, 35, 38–41, 43]. However, these crude event counts were not directly comparable because they were influenced by differences in follow-up duration, censoring, and survival data maturity across studies.

Given the heterogeneity in reporting and the absence of consistently extractable time-to-event effect estimates, a formal quantitative synthesis of OS was not performed. Instead, OS findings were summarized narratively. Across the available studies, survival outcomes appeared generally encouraging within the reported follow-up periods, although interpretation remains limited by inconsistent endpoint reporting and relatively immature long-term survival data in several cohorts.

### Progression-free survival (PFS)

Progression-free survival (PFS) was infrequently reported across the included studies. Only three studies reported progression events during follow-up, with Li et al. [40] Sun et al. [41], and Wang et al. [33] reporting 4, 15, and 9 progression events, respectively. However, these event counts were not directly comparable because PFS is a time-to-event endpoint and is influenced by differences in follow-up duration, censoring, and the maturity of survival reporting.

Given the limited number of studies and the lack of consistently extractable time-to-event estimates, a formal quantitative synthesis of PFS was not performed. Accordingly, PFS outcomes were summarized narratively. Although progression events were observed across available cohorts, the current evidence remains insufficient to support a reliable pooled estimate of progression-free survival after neoadjuvant immunotherapy.

### Disease-free survival (DFS) and recurrence

Disease-free survival (DFS) outcomes were reported inconsistently across the included studies and were primarily described through recurrence events during follow-up rather than through standardized time-to-event estimates. Seven studies reported recurrence, with Guo et al. [31], Jiang et al. [38], Li et al. [39], Pan et al. [32], Verschoor et al. [43], Wang et al. [33], and Xu et al. [35] reporting 4, 5, 5, 9, 7, 9, and 41 recurrence events, respectively. However, these recurrence counts were not directly comparable across studies because DFS is a time-to-event endpoint and is influenced by differences in follow-up duration, censoring, and the maturity of survival reporting.

Given the heterogeneity in outcome definitions and the absence of consistently extractable time-to-event effect estimates, a formal quantitative synthesis of DFS was not performed. Instead, DFS and recurrence findings were summarized narratively. Although recurrence events were documented across several cohorts, the currently available evidence remains insufficient to support a robust pooled estimate of disease-free survival following neoadjuvant immunotherapy-based treatment.

#### Event-free survival (EFS)

Event-free survival (EFS) was sparsely reported across the included studies. Only two studies provided information on event occurrence during follow-up, with Li et al. [39] and Shitara et al. [29] reporting 8 and 194 events, respectively. However, these event counts were not directly comparable because EFS is a time-to-event endpoint and is influenced by differences in follow-up duration, censoring, study size, and the maturity of survival reporting.

Given the very limited number of studies and the absence of consistently extractable time-to-event estimates, a formal quantitative synthesis of EFS was not performed. Accordingly, EFS findings were summarized narratively, and the currently available data remain insufficient to support robust conclusions regarding event-free survival after neoadjuvant immunotherapy-based treatment.

### Subgroup analysis

Subgroup analyses were performed to examine whether pathologic response varied according to neoadjuvant regimen intensity and study design. When studies were stratified by regimen intensity, the pooled pCR rate was 22% (95% CI, 17%–27%; I² = 63.2%) in the chemotherapy-only subgroup and 26% (95% CI, 18%–38%; I² = 75.0%) in the intensified regimen subgroup (Supplementary Fig. 1). The corresponding pooled MPR rates were 47% (95% CI, 40%–54%; I² = 68.1%) and 53% (95% CI, 41%–66%; I² = 79.0%) (Supplementary Fig. 2), respectively. Although point estimates were numerically higher in the intensified subgroup, the between-subgroup differences were not statistically significant for either pCR (*p* = 0.3869) or MPR (*p* = 0.3733).

When studies were stratified by study design, the pooled pCR rate was 21% (95% CI, 17%–25%; I² = 57.3%) in comparative studies and 27% (95% CI, 18%–38%; I² = 69.2%) in single-arm studies (Supplementary Fig. 3). For MPR, the pooled estimate was 47% (95% CI, 39%–55%; I² = 79.3%) in comparative studies and 53% (95% CI, 43%–63%; I² = 64.2%) in single-arm studies (Supplementary Fig. 4). Again, although single-arm studies showed numerically higher pooled estimates, the subgroup differences were not statistically significant for either pCR (*p* = 0.2537) or MPR (*p* = 0.3404).

When studies were stratified according to treatment strategy, the pooled pCR rate was 22% (95% CI, 18%–28%; I² = 44.2%) among neoadjuvant-only studies and 24% (95% CI, 18%–32%; I² = 79.5%) among perioperative studies (Supplementary Fig. 5). No statistically significant difference was observed between treatment strategies for pCR (*p* = 0.6598), indicating that pCR rates were broadly comparable across neoadjuvant-only and perioperative approaches. Similarly, for MPR, the pooled rate was 45% (95% CI, 34%–56%; I² = 81.1%) among neoadjuvant-only studies and 53% (95% CI, 47%–59%; I² = 34.4%) among perioperative studies (Supplementary Fig. 6). Although the pooled MPR estimate was numerically higher in the perioperative subgroup, the between-subgroup difference was not statistically significant (*p* = 0.1796).

Overall, subgroup analyses according to regimen intensity, study design, and treatment strategy showed no statistically significant differences in pCR or MPR, suggesting that the pooled pathologic response findings were generally consistent across these stratifications.

**Table 3:** Subgroup Analyses of Pathologic Response According to Regimen Intensity, Study Design and Treatment Strategy

Subgroup variable	Outcome	Subgroup	Pooled estimate (95% CI)	I² (%)	*p* for subgroup difference
Regimen intensity	pCR	Chemotherapy-only	22% (17%–27%)	63.2	0.3869
		Intensified regimen	26% (18%–38%)	75.0	
	MPR	Chemotherapy-only	47% (40%–54%)	68.1	0.3733
		Intensified regimen	53% (41%–66%)	79.0	
Study design	pCR	Comparative studies	21% (17%–25%)	57.3	0.2537
		Single-arm studies	27% (18%–38%)	69.2	
	MPR	Comparative studies	47% (39%–55%)	79.3	0.3404
		Single-arm studies	53% (43%–63%)	64.2	
Treatment strategy	pCR	Neoadjuvant-only	22% (18%–28%)	44.2	0.6598
		Perioperative	24% (18%–32%)	79.5	
	MPR	Neoadjuvant-only	45% (34%–56%)	81.1	0.1796
		Perioperative	53% (47%–59%)	34.4	

### Sensitivity analysis

Leave-one-out sensitivity analyses were performed for both pCR and MPR to assess the robustness of the pooled estimates. For pCR, sequential omission of individual studies did not materially change the pooled effect size, with pooled estimates remaining within a narrow range of 22%–24% and without any meaningful reduction in between-study heterogeneity (Supplementary Fig. 7). Similarly, for MPR, exclusion of each study in turn produced pooled estimates ranging from 48% to 51%, again without substantial deviation from the overall pooled estimate or notable change in heterogeneity (Supplementary Fig. 8). These findings indicate that the pooled pCR and MPR results were stable and were not disproportionately influenced by any single study.

### Publication bias

**Fig. 11:**
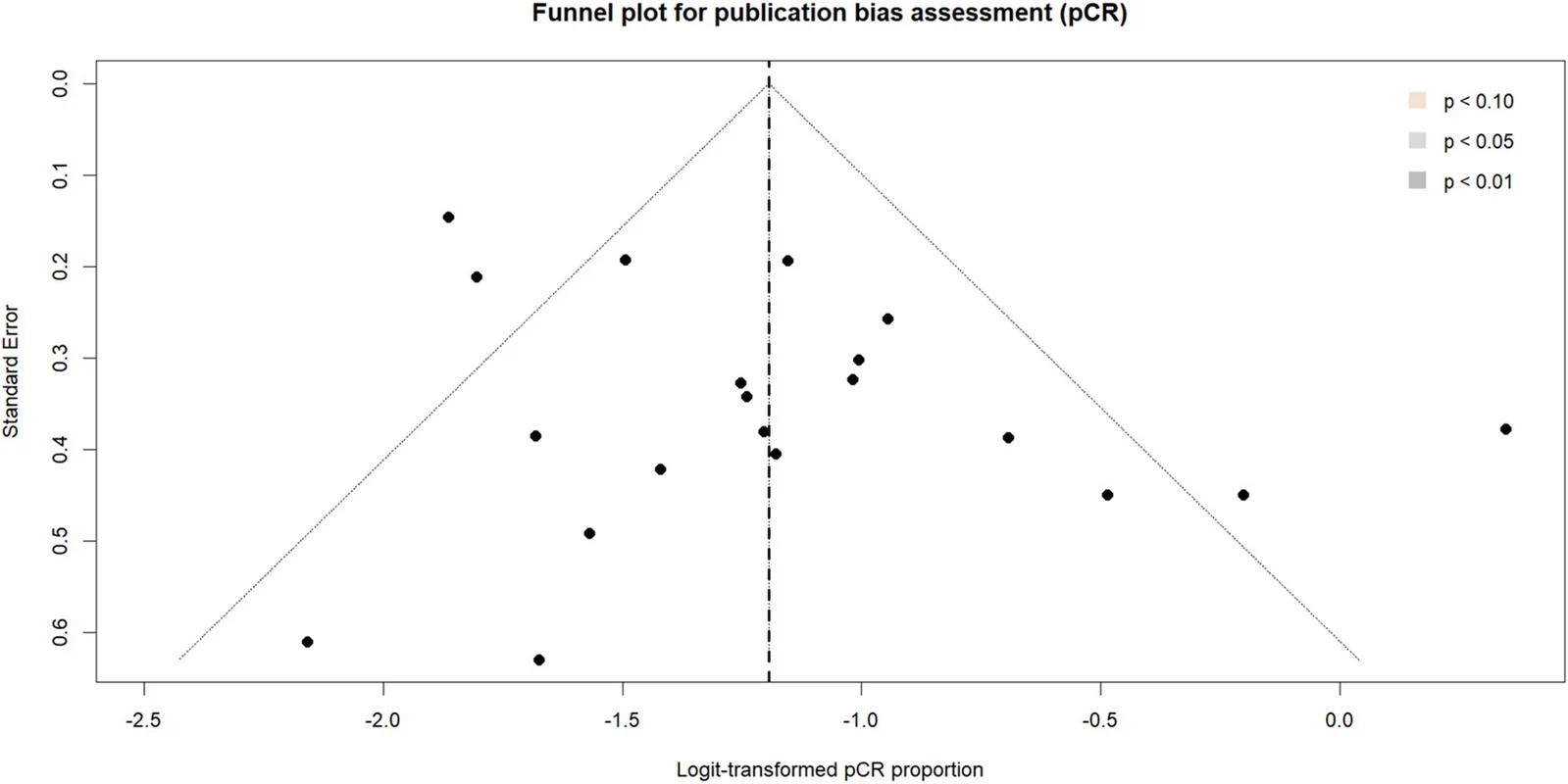
Funnel plot assessing publication bias for pathologic complete response (pCR)

**Fig. 12:**
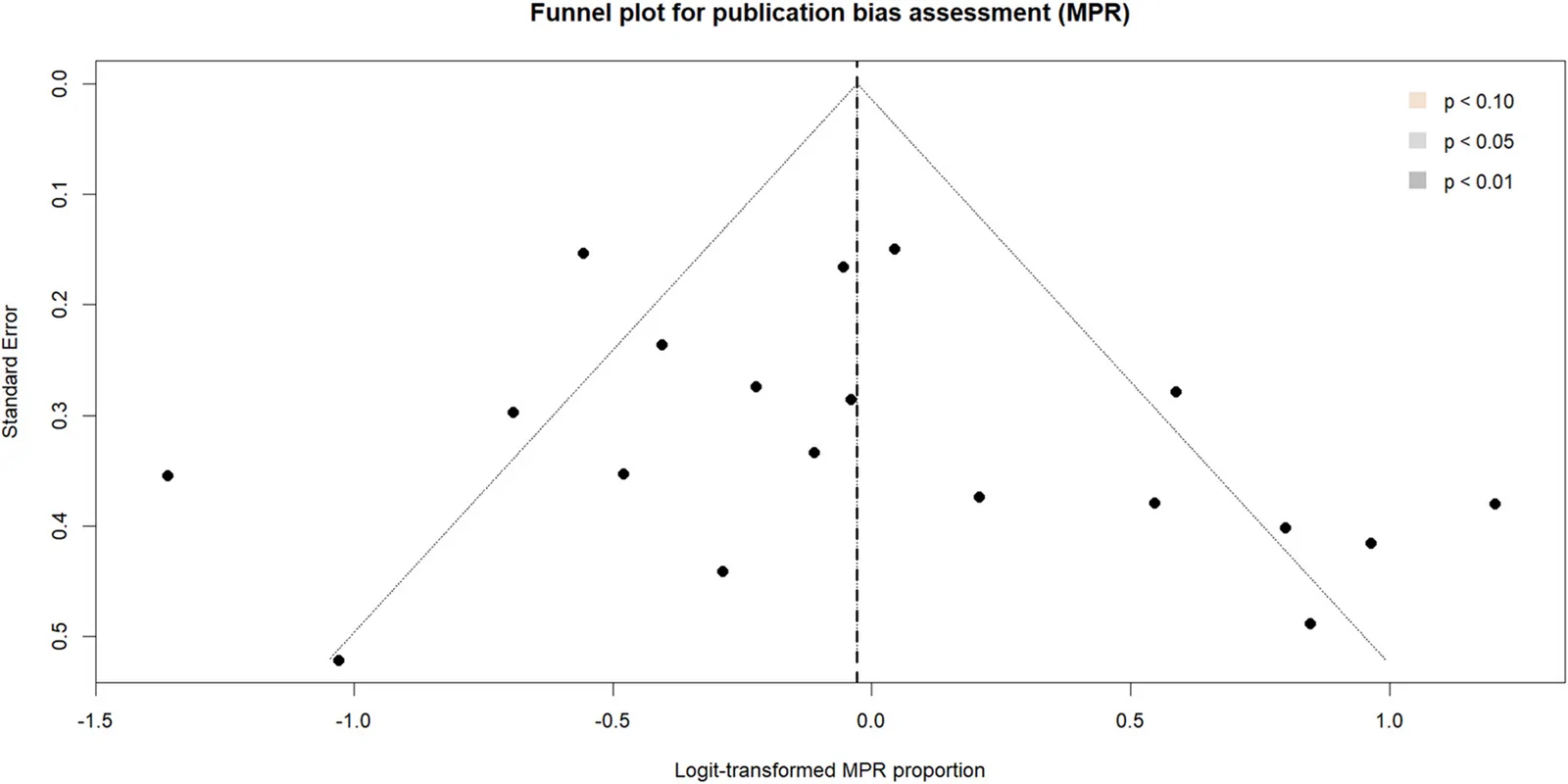
Funnel plot assessing publication bias for major pathologic response (MPR)

The Assessment of publication bias showed mixed findings. For pCR, visual inspection of the funnel plot suggested mild asymmetry, and Egger’s regression test indicated potential small-study effects (*p* = 0.0439). However, Begg’s rank correlation test was not statistically significant (*p* = 0.6970). In contrast, for MPR, the funnel plot appeared relatively symmetric, and neither Egger’s regression test (*p* = 0.3042) nor Begg’s rank correlation test (*p* = 0.2781) showed significant evidence of publication bias. Overall, these findings suggest that possible publication bias may be present for pCR, whereas no clear evidence of publication bias was observed for MPR (Figs. 11and 12).

## Discussion

This systematic review and meta-analysis demonstrate that neoadjuvant immune checkpoint inhibitor (ICI)-based therapy is associated with encouraging pathological response and high rates of curative-intent resection in patients with locally advanced resectable gastric and gastroesophageal junction (GEJ) adenocarcinoma. Across 20 studies, the pooled pathologic complete response (pCR) rate was 22.8%, the pooled major pathologic response (MPR) rate was 49.5%, the pooled objective response rate (ORR) was 60.6%, and the pooled R0 resection rate was 97.2%. These findings suggest that adding immunotherapy to preoperative treatment can achieve meaningful tumor regression while maintaining surgical feasibility in appropriately selected patients.

The pathological activity observed in the present study is clinically important. In resectable gastric and GEJ adenocarcinoma, pCR and MPR are useful early indicators of treatment effect because they directly reflect tumor sensitivity to neoadjuvant therapy and can be assessed before mature survival data become available. Several prospective and retrospective studies have reported favorable tumor regression with PD-1/PD-L1-based combinations, including sintilimab-, camrelizumab-, atezolizumab-, and toripalimab-containing regimens [26, 27, 30, 31, 37, 38, 41–43]. The pooled MPR rate approaching 50% is particularly notable, as major pathological regression may be a more sensitive marker of treatment activity than pCR alone in gastric cancer, where complete pathological eradication is less common. In addition, the stability of MPR on leave-one-out sensitivity analysis supports the robustness of this signal despite between-study heterogeneity.

Although these pathological response rates are encouraging, they should be interpreted as early indicators of treatment activity rather than evidence of durable oncologic benefit. Mature and consistently reported event-free survival, disease-free survival, and overall survival data remain limited across the available studies. Consequently, the present findings should be viewed as supportive of continued investigation rather than sufficient to establish a change in current clinical practice.

Recent pivotal randomized trials have further shaped the interpretation of pathological response outcomes in this setting. Both MATTERHORN and KEYNOTE-585 demonstrated that the addition of immunotherapy to perioperative treatment can improve pathological response measures, supporting the biological activity of immune checkpoint blockade in resectable gastric and gastroesophageal junction adenocarcinoma [29, 43]. However, the final analysis of KEYNOTE-585 also demonstrated that improvements in pathological response do not necessarily translate into statistically significant overall survival benefits in unselected populations [29]. Therefore, although the favorable pCR and MPR rates observed in the present meta-analysis are encouraging, these endpoints should be interpreted cautiously. Evidence from KEYNOTE-585 demonstrates that improvements in pathological response do not necessarily translate into significant overall survival benefit in unselected populations, indicating that pCR and MPR should not be regarded as definitive surrogate endpoints for long-term oncologic outcomes. Mature survival data from ongoing randomized studies remain essential to determine the clinical significance of these pathological responses.

Importantly, the comparative evidence from randomized and controlled studies should be considered separately from the pooled proportion estimates presented in this meta-analysis. Whereas the pooled analyses describe pathological response and surgical outcomes observed after ICI-based treatment across diverse study populations, randomized trials provide the most appropriate evidence for determining whether these approaches offer additional benefit over current perioperative chemotherapy. The available comparative studies generally support the biological activity of immunotherapy; however, the magnitude of clinical benefit has not been uniform across trials, reinforcing the need to interpret descriptive pooled response rates separately from comparative treatment effects.

The high pooled R0 resection rate is equally relevant from a surgical perspective. Intensification of preoperative therapy raises concerns regarding treatment-related toxicity, delay to surgery, and the potential loss of resectability. In the present analysis, R0 resection remained high, indicating that ICI-based strategies can generally be delivered without compromising operability. This is consistent with prospective and randomized studies in which surgery after neoadjuvant or perioperative immunotherapy was feasible within multidisciplinary treatment pathways [26, 27, 30, 37, 43]. Although grade ≥ 3 adverse events, immune-related adverse events, and postoperative complications were generally manageable, the available evidence suggests that ICI-based treatment did not preclude surgical resection in the included studies. However, interpretation of safety outcomes should remain cautious because definitions of postoperative complications, surgical morbidity reporting, and toxicity assessment varied across studies. Furthermore, because many included studies lacked comparator arms, it remains uncertain whether the observed safety outcomes are superior, equivalent, or inferior to those associated with contemporary perioperative chemotherapy. Consequently, definitive conclusions regarding the comparative safety of ICI-based strategies cannot be drawn from the current evidence.

Our findings are broadly consistent with previous systematic reviews and meta-analyses, which have also reported encouraging pathological response with neoadjuvant immunotherapy in resectable gastric and GEJ cancer [12–14]. However, the present study provides an independent synthesis using a broader and more contemporary evidence base, incorporating recent phase II studies, randomized trials, and newly reported 2025 data. This is relevant because the evidence in this field has evolved rapidly, and earlier reviews included fewer prospective comparative studies and less mature perioperative immunotherapy data [27, 28, 30, 32, 43].

At the same time, the present findings should be interpreted with considerable caution because of the substantial clinical heterogeneity across included studies. Importantly, the pooled analyses combined several distinct therapeutic approaches, including ICI plus chemotherapy, ICI plus antiangiogenic therapy, ICI plus HER2-directed therapy, chemotherapy-free dual immune checkpoint blockade, and perioperative regimens with differing postoperative treatment exposures. In neoadjuvant-only studies, immunotherapy-based treatment was completed before surgery, whereas perioperative strategies also incorporated planned postoperative systemic therapy. This distinction is clinically important because postoperative treatment may independently influence recurrence and survival outcomes, making it difficult to attribute longer-term outcomes solely to the preoperative component of treatment. Because postoperative chemotherapy and/or adjuvant immunotherapy may contribute to long-term outcomes, the effects observed for survival endpoints cannot be attributed solely to the neoadjuvant component of treatment. This consideration further supports the decision to summarize survival outcomes narratively rather than perform a pooled survival analysis. In addition, the included studies differed with respect to treatment timing, with some evaluating neoadjuvant therapy alone and others employing perioperative strategies that incorporated postoperative chemotherapy, immunotherapy, targeted therapy, or maintenance treatment. These differences may be particularly relevant when interpreting survival outcomes, as postoperative treatment exposure could contribute to long-term efficacy independently of the neoadjuvant component. These approaches are biologically and clinically different and may not be expected to produce equivalent efficacy or safety outcomes. Therefore, the pooled estimates should not be interpreted as representing the effect of a single treatment strategy. Rather, they reflect the aggregate activity of a broad group of ICI-containing preoperative approaches. The substantial heterogeneity observed across several outcomes, including pCR, MPR, ORR, and safety endpoints, likely reflects these important differences in regimen composition, patient selection, and treatment intensity [25, 28, 33, 34, 37, 40]. This clinical diversity was also reflected in the statistical heterogeneity observed across several pooled analyses, with moderate to high I² values identified for key efficacy and safety outcomes. Such heterogeneity indicates that the magnitude of treatment effects varied considerably across studies and further supports cautious interpretation of the pooled estimates.

Although pooling these studies provides an overall estimate of the activity of ICI-based preoperative treatment, it does not imply that these therapeutic approaches are clinically equivalent. Regimens combining ICIs with chemotherapy, antiangiogenic agents, HER2-directed therapy, or dual immune checkpoint blockade differ in their biological rationale, mechanisms of action, and toxicity profiles. Therefore, the pooled estimates should be interpreted as representing the overall activity of heterogeneous ICI-based strategies rather than the effect of any individual treatment approach.

Importantly, subgroup analyses showed numerically higher pCR and MPR rates with intensified regimens, but no statistically significant subgroup differences were detected for regimen intensity, study design, or treatment strategy (neoadjuvant-only versus perioperative), suggesting that the overall pathological response signal was not restricted to a single treatment platform or study type. However, these subgroup analyses were exploratory and were unable to fully account for the marked clinical diversity of the included regimens. Therefore, residual heterogeneity remains an important limitation when interpreting the pooled results. Although subgroup analyses according to regimen intensity, study design, and treatment strategy were performed, these analyses did not identify significant differences between subgroups, suggesting that the observed heterogeneity likely reflects multiple overlapping clinical and methodological factors that could not be fully captured using study-level data. Consequently, the subgroup analyses should be regarded as exploratory and should not be interpreted as demonstrating equivalence between the different therapeutic strategies evaluated.

Furthermore, the regimen-intensity classification used in the present study may not fully capture clinically relevant differences among treatment backbones. Intensified regimens included heterogeneous approaches such as antiangiogenic combinations, HER2-directed strategies, and dual immune checkpoint blockade, each of which may have distinct efficacy and safety profiles. Owing to the limited number of studies within several treatment categories, particularly HER2-targeted therapy and dual immune checkpoint blockade, more granular subgroup analyses were not considered statistically robust. Consequently, potentially important differences between specific treatment platforms may have been obscured in the pooled estimates. This limitation further highlights that the results of the present meta-analysis should be interpreted as a broad overview of current ICI-based preoperative strategies rather than evidence supporting any specific regimen.

From a clinical perspective, these subgroup findings suggest that treatment intensity alone is unlikely to determine the effectiveness of perioperative immunotherapy. Instead, treatment outcomes probably reflect the combined influence of regimen composition, patient selection, tumour biology, and postoperative treatment. Therefore, the observed subgroup differences should be considered exploratory and may help generate hypotheses for future trials rather than guide treatment selection in current clinical practice.

Although an additional subgroup analysis according to treatment strategy (neoadjuvant-only versus perioperative) was performed, more granular analyses according to individual immune checkpoint inhibitors or tumor location (gastric versus gastroesophageal junction adenocarcinoma) were not feasible because these data were inconsistently reported and several treatment categories were represented by only a small number of studies.

Another important limitation is that the current evidence base remains dominated by single-arm studies and relatively small cohorts, with only a limited number of randomized or comparative datasets. Several pooled analyses also demonstrated moderate-to-substantial statistical heterogeneity, indicating considerable variability in treatment effects across studies. High statistical heterogeneity reduces confidence in the precision and generalizability of the pooled estimates because the observed treatment effects were not consistent across studies. Accordingly, the summary estimates should be interpreted as average effects across diverse study populations and treatment approaches rather than as precise estimates applicable to all clinical settings. Although some of this variability likely reflects differences in treatment regimens, study design, and patient populations, it could not be fully explained by the available subgroup analyses. Together, these factors increase the risk of bias and contribute to uncertainty in the pooled estimates, particularly for pathological endpoints. The predominance of non-comparative studies also limits the interpretation of safety findings, as direct comparisons with current perioperative chemotherapy standards were unavailable in many datasets. Another limitation is the predominance of Asian cohorts, particularly from China, which may limit the generalizability of the findings to Western populations with different patient characteristics, treatment practices, and tumor biology. Publication bias must also be considered. In the present study, Egger’s test suggested possible small-study effects for pCR, whereas no clear evidence of publication bias was observed for MPR by either Egger’s or Begg’s testing. This pattern is plausible in a rapidly expanding field where smaller positive studies may be preferentially published, especially when pCR is emphasized as a key endpoint. However, the absence of a similar signal for MPR, together with stable sensitivity analyses, suggests that the overall findings are unlikely to be explained solely by publication bias.

An additional consideration is that most pooled efficacy outcomes in the present meta-analysis were derived from single-arm studies. Although meta-analysis of proportions provides useful summary estimates of pathological response, radiological response, and surgical outcomes following ICI-based treatment, these pooled estimates should not be interpreted as evidence that ICI-based strategies are superior to contemporary perioperative chemotherapy. Direct comparisons regarding treatment effectiveness require adequately powered randomized or comparative studies.

A further limitation is the immaturity and inconsistency of survival reporting. Although several included studies reported overall survival, disease-free survival, progression-free survival, or event-free survival, these outcomes were inconsistently defined and incompletely reported, which precluded reliable pooled time-to-event analysis. As a result, the present study appropriately focused on pathological, radiological, and surgical outcomes, while survival endpoints were summarized narratively. Consequently, no definitive conclusions regarding overall survival benefit can be drawn from the current evidence base. This is a key issue because pathological response, although clinically informative, cannot substitute for durable long-term benefit in a disease where recurrence remains common after apparently curative treatment. Consequently, the efficacy findings of the present meta-analysis should be regarded as hypothesis-generating rather than practice-changing until supported by mature survival data from adequately powered randomized trials. In addition, this review was not prospectively registered in PROSPERO. Although the methodology was defined a priori, the absence of prospective registration may reduce transparency and should be considered when interpreting the findings.

Recent randomized evidence further reinforces the need for cautious interpretation of pathological response outcomes. The final analysis of KEYNOTE-585 demonstrated improved pathological response with perioperative pembrolizumab-based therapy but did not show a statistically significant overall survival benefit in the overall study population [29]. In contrast, MATTERHORN reported encouraging improvements in pathological response and event-free survival outcomes, although longer follow-up remains necessary to fully establish the durability of benefit [43]. Together, these randomized studies indicate that pathological response is an important measure of antitumor activity but remains an imperfect surrogate for long-term oncologic outcomes. Consequently, improvements in pCR or MPR should be interpreted alongside mature survival data when assessing the clinical benefit of perioperative immunotherapy. In parallel, biomarker-enriched strategies have shown particularly strong activity in selected populations, including dMMR/MSI-H disease in NEONIPIGA and HER2-positive disease in recent perioperative combination studies [28, 37]. These observations support a more individualized approach to immunotherapy, with treatment selection guided by tumor biology rather than a uniform strategy for all patients. This growing emphasis on biomarker-guided treatment may help maximize clinical benefit while avoiding unnecessary treatment in patients less likely to respond.

Another important consideration is the growing role of biomarker-guided treatment selection in optimizing the use of perioperative immunotherapy. Evidence from biomarker-enriched cohorts suggests that the benefit of immunotherapy may not be uniform across all resectable gastric and GEJ adenocarcinomas. Patients with dMMR/MSI-H tumors have demonstrated particularly high pathological response rates with immune checkpoint blockade, including chemotherapy-free approaches, supporting the exceptional immunogenicity of this molecular subtype [37]. Similarly, HER2-positive disease has shown promising activity with combined HER2-targeted and immunotherapy-based perioperative strategies [28]. PD-L1 expression may also contribute to treatment stratification, although its predictive value in the perioperative setting remains less clearly established and requires further validation. Collectively, these findings support an evolving treatment paradigm in which the benefit of perioperative immunotherapy appears to be biologically enriched rather than uniform across all patients with resectable gastric and gastroesophageal junction adenocarcinoma. Future treatment strategies are therefore likely to rely increasingly on molecular and immunologic biomarkers to guide patient selection.

From a clinical perspective, these data support continued evaluation of ICI-based neoadjuvant and perioperative strategies, but they do not yet establish a universal standard of care for all resectable gastric and GEJ adenocarcinomas. Perioperative chemotherapy remains the reference approach in many settings, particularly with FLOT-based treatment [3]. The future direction of this field will likely depend on better biological selection, standardized pathological reporting, and more mature randomized survival data. In particular, future trials should prioritize biomarker-driven analyses, especially for MSI/dMMR, PD-L1 expression, and HER2 status, as well as clearer reporting of postoperative morbidity and long-term oncologic outcomes.

Overall, the present meta-analysis shows that neoadjuvant or perioperative ICI-based therapy is associated with meaningful pathological regression and high rates of R0 resection in locally advanced resectable gastric and GEJ adenocarcinoma. The short-term efficacy signal is encouraging, and the available surgical outcomes appear acceptable. However, these findings should be interpreted cautiously given the absence of mature and consistently reported survival outcomes across studies.

## Conclusion

Neoadjuvant or perioperative immune checkpoint inhibitor-based therapy is associated with encouraging pathological response and high R0 resection rates in patients with locally advanced resectable gastric and gastroesophageal junction adenocarcinoma. These findings support the feasibility and short-term antitumor activity of ICI-containing preoperative strategies. However, because much of the available evidence was derived from single-arm studies, these pooled efficacy estimates describe outcomes following ICI-based treatment rather than demonstrating superiority over current standards of perioperative chemotherapy. However, the current evidence remains substantially limited by marked clinical heterogeneity across treatment strategies, including differences in systemic treatment backbones, targeted therapy combinations, dual-checkpoint approaches, and postoperative treatment exposure. Consequently, the pooled estimates should be interpreted as reflecting the overall activity of heterogeneous ICI-based preoperative strategies rather than the effectiveness of any single therapeutic approach. Because the included regimens differ substantially in their treatment backbone, biological mechanisms, and postoperative management, direct comparisons between individual strategies cannot be made from this meta-analysis. Additional limitations include immature survival data and the predominance of non-comparative evidence, which preclude definitive conclusions regarding long-term survival benefit. Further large randomized trials with mature survival outcomes and biomarker-guided analyses are needed to better define the patients most likely to benefit from these approaches and to establish the role of perioperative immunotherapy within biologically selected patient populations rather than as a universal treatment strategy for all resectable gastric and gastroesophageal junction adenocarcinomas.

## Supplementary Information

Supplementary Material 1.

## Data Availability

All data analysed during this study are included in this published article and its supplementary information. The datasets are derived from previously published studies, which are cited in the reference list. Additional details are available from the corresponding author upon reasonable request.
