# Supplementary material for: Neoadjuvant immunotherapy for locally advanced resectable gastric and gastroesophageal junction adenocarcinoma: a systematic review and meta-analysis
Source: BMC Surg. 2026 Jul 29;26:719. doi: 10.1186/s12893-026-04048-y (PMC13700275; doi:10.1186/s12893-026-04048-y)
Supplement: Supplementary file 1 [file 12893_2026_4048_MOESM1_ESM.docx]

**Appendix**

**Table 2. Characteristics of Included Studies**

| Study, Design | Country | Sample size (ICI cohort) | Tumor Site | Treatment Strategy | Neoadjuvant ICI-based Regimen | Outcomes Reported | Key findings |
| --- | --- | --- | --- | --- | --- | --- | --- |
| (Guo et al., 2022), Retrospective, comparative cohort study. | China | 30 patients | Gastric adenocarcinoma (locally advanced, resectable) | Perioperative (4 cycles neoadjuvant + surgery + 4 cycles adjuvant) | Sintilimab (anti–PD-1) + XELOX (oxaliplatin + capecitabine) | pCR, MPR, ORR, DCR, R0 resection rate, TNM downstaging, DFS, OS, surgical complications, irAEs, Grade ≥3 TRAEs | High pCR (33.3%) and MPR (63.3%) with 100% R0 resection; manageable toxicity and acceptable surgical safety |
| (Jiang et al., 2022), Prospective single-arm | China | 36 patients | Gastric adenocarcinoma (80.6%) and GEJ adenocarcinoma (19.4%) | Neoadjuvant | Sintilimab (anti–PD-1) + CapeOx (oxaliplatin + capecitabine) | pCR, MPR, ORR, TNM downstaging, R0 resection rate, DFS, OS, surgical complications, irAEs, Grade ≥3 TRAEs | pCR 19.4%, MPR 47.2%, R0 resection 97.2%; encouraging efficacy with manageable toxicity and high 1-year DFS/OS rates |
| (Li et al., 2024), single-arm prospective cohort study | China | 32 patients | Gastric adenocarcinoma (81.3%) and GEJ adenocarcinoma (18.7%) | Perioperative (3 cycles sintilimab + 4 cycles FLOT pre-op, surgery, then 4 cycles adjuvant FLOT) | Sintilimab (anti–PD-1) + FLOT (fluorouracil, leucovorin, oxaliplatin, docetaxel) | pCR, MPR, ORR, DCR, R0 resection rate, TNM downstaging, DFS, OS, EFS, surgical complications, irAEs, Grade ≥3 TRAEs | pCR 17.2%, MPR 55.2%, R0 resection 93.1%; 3-year DFS 78.8%, OS 70.9%; manageable toxicity, no grade ≥3 surgical complications |
| (Li et al., 2023), Prospective, single‑arm study | China | 25 patients enrolled; 19 evaluable for pathological response | Gastric adenocarcinoma (locally advanced, cT4a/bN+) | Neoadjuvant/conversion | Camrelizumab (anti–PD-1) + apatinib (VEGFR2 TKI) + S-1 (± oxaliplatin) | pCR, MPR, PPR, ORR, tumor downstaging, R0 resection rate, DFS, OS, surgical complications, irAEs, Grade ≥3 TRAEs | pCR 15.8%, MPR 26.3%, R0 resection 82.6%; high downstaging rate (76%) with limited toxicity and acceptable surgical safety |
| (Lin et al., 2024), Multicenter, RCT | China | 51 patients | Gastric adenocarcinoma (clinical T2–4N+M0) | Neoadjuvant | Camrelizumab (anti–PD-1) + apatinib (VEGFR2 TKI) + nab-paclitaxel + S-1 | pCR, MPR, ORR, DCR, R0 resection rate, TNM downstaging, surgical complications, irAEs, Grade ≥3 TRAEs | MPR 33.3%, pCR 15.7%, ORR 66%, R0 resection 94.1%; toxicity manageable, surgical safety acceptable |
| (Pan et al., 2025), Single-center, retrospective real-world study | China | 49 patients received PD-1 inhibitor + chemotherapy | Gastric adenocarcinoma (95.5%) and GEJ adenocarcinoma (small proportion) | Neoadjuvant | PD-1 inhibitors (sintilimab, tislelizumab, nivolumab, toripalimab, pembrolizumab) + chemotherapy (SOX, CapeOx, nab-paclitaxel + S-1) | pCR, MPR, TNM downstaging, R0 resection rate, DFS, OS, surgical complications, irAEs, Grade ≥3 TRAEs | pCR 22.4% vs 4.8% (chemo alone), 100% R0 resection; toxicity manageable, no excess surgical risk |
| (Sun et al., 2024), Prospective, single‑arm | China | 49 patients | Gastric adenocarcinoma (75.5%) and GEJ adenocarcinoma (24.5%) | Perioperative (3 cycles neoadjuvant + surgery + 5 cycles adjuvant SOX) | Tislelizumab (anti–PD‑1) + SOX (S‑1 + oxaliplatin) | pCR, MPR, R0 resection rate, TNM downstaging, PFS, OS, surgical complications, irAEs, Grade ≥3 TRAEs | pCR 26.5%, MPR 49.0%, R0 resection 100%; 2‑year PFS 69.4%, OS 81.2%; toxicity acceptable, one fatal irAE (HLH) |
| (Tang et al., 2022), Retrospective, single‑arm observational study | China | 75 patients | Gastric adenocarcinoma (72.5%) and GEJ adenocarcinoma (27.5% | Neoadjuvant | Camrelizumab (anti–PD‑1) + FOLFOX (oxaliplatin + leucovorin + 5‑FU) | pCR, MPR, ORR, R0 resection rate, TNM downstaging, DFS, OS, surgical complications, irAEs, Grade ≥3 TRAEs | pCR 20%, MPR 45%, R0 resection 95%; encouraging efficacy with acceptable toxicity and surgical safety |
| (Verschoor et al., 2024), Prospective, single‑arm | Netherlands | 21 patients enrolled; 20 evaluable for efficacy | Gastric adenocarcinoma (24%) and GEJ adenocarcinoma (76%) | Pure neoadjuvant | Atezolizumab (anti–PD‑L1) + docetaxel + oxaliplatin + capecitabine | pCR, MPR, R0 resection rate, DFS, OS, surgical complications, irAEs, Grade ≥3 TRAEs | MPR 70%, pCR 45%, R0 resection 95%; excellent correlation between pathologic response and survival, manageable toxicity |
| (Wang et al., 2023), Prospective, non‑randomized cohort study | China | 39 patients | Gastric adenocarcinoma (76.9%) and GEJ adenocarcinoma (23.1%) | Neoadjuvant | PD‑1 inhibitors (sintilimab, camrelizumab, or toripalimab) + apatinib + SOX/CapeO | pCR, pathologic response, ORR, DCR, R0 resection rate, DFS, PFS, OS, surgical complications, irAEs, Grade ≥3 TRAEs | PAC group showed higher ORR (74.4%), pCR (23.1%), R0 resection (97.4%), and prolonged 3‑year PFS (76.1%) and OS (86.7%) compared to AC; toxicity manageable, no excess surgical risk |
| (Xiong & Li, 2023), Retrospective, comparative cohort study | China | 56 patients | Gastric adenocarcinoma (76.8%) and GEJ adenocarcinoma (23.2%) | Neoadjuvant | Sintilimab or camrelizumab (PD‑1 inhibitor) + apatinib + SOX/CapeOx | ORR, DCR, pCR, pathological response, R0 resection rate, PFS, DFS, OS, adverse events | PAC group achieved higher ORR (73.2%), pCR (26.8%), and longer PFS/OS vs chemo alone; toxicity acceptable, peripheral neuropathy slightly increased |
| (Xu et al., 2024), Multicenter, retrospective, real‑world cohort study | China | 201 patients received ICIs + chemotherapy (184 matched for analysis) | Gastric adenocarcinoma and GEJ adenocarcinoma | Neoadjuvant | Anti‑PD‑1/PD‑L1 ICIs (various agents) + doublet/triplet chemotherapy (SOX, XELOX, FLOT, DOS, DCF, DCX) | pCR, ORR, DFS, OS, R0 resection rate, surgical complication rate | ICIs + chemo improved pCR (14.1% vs 7.6%) and 2–3 year DFS, with no excess surgical complications; OS benefit not significant |
| (Zhang et al., 2023), Retrospective, single‑center comparative cohort study | China | 34 patients | Gastric adenocarcinoma (76.5%) and GEJ adenocarcinoma (23.5%) | Neoadjuvant | Camrelizumab, sintilimab, nivolumab, or toripalimab + FLOT or SOX | pCR, MPR, ORR, R0 resection rate, TNM downstaging, RFS, OS, surgical complications, Grade ≥3 TRAEs | pCR 23.5% vs 4.7% (chemo), MPR 38.2%, R0 resection 97.1%; improved pCR with acceptable toxicity |
| (Zhong et al., 2024), Prospective, single‑arm study | China | 29 patients | Gastric adenocarcinoma (41.4%) and GEJ adenocarcinoma (58.6%) | Perioperative (3 cycles neoadjuvant + surgery + 3 cycles adjuvant) | Camrelizumab (anti–PD‑1) + oxaliplatin + S‑1 (SOX regimen) | pCR, tpCR, MPR, ORR, DCR, R0 resection rate, TNM downstaging, EFS, OS, surgical complications, irAEs, Grade ≥3 TRAEs | pCR 10.3%, MPR 69%, R0 resection 96.6%; modest pCR but high MPR with excellent safety (no grade ≥3 AEs) |
| (André et al., 2023), Prospective, single‑arm study | France | 32 patients | Gastric adenocarcinoma (50%) and GEJ adenocarcinoma (50%) | Perioperative (12 weeks neoadjuvant nivolumab + ipilimumab → surgery → adjuvant nivolumab for 9 cycles) | Nivolumab (anti–PD‑1) + ipilimumab (anti–CTLA‑4) | pCR, R0 resection rate, EFS, OS, surgical complication rate, Grade ≥3 TRAEs | pCR 59%, R0 resection 100%; high response rates with manageable toxicity, though surgical morbidity was notable |
| (Li et al., 2025), Multicenter, RCT | China | 180 patients | Gastric adenocarcinoma (69%) and GEJ adenocarcinoma (31%) | Perioperative (3 cycles neoadjuvant → surgery → 3 cycles adjuvant + extended camrelizumab/rivoceranib maintenance) | Camrelizumab (anti–PD‑1) + low‑dose rivoceranib (VEGFR2 TKI) + SOX (S‑1 + oxaliplatin) | pCR, MPR, total pCR (ypT0N0), R0 resection rate, surgical complication rate, irAEs, Grade ≥3 TRAEs, EFS (ongoing) | pCR 18.3% vs 5.0% (SOX), MPR 51.1% vs 37.8%, R0 resection 99% vs 94%; improved pathologic response with manageable toxicity |
| (Lorenzen et al., 2023), Multicenter, RCT | Germany & Switzerland | 146 patients | Gastric adenocarcinoma (38%) and GEJ adenocarcinoma (62%) | Perioperative (4 cycles neoadjuvant + surgery + 4 cycles adjuvant + atezolizumab maintenance) | Atezolizumab (anti–PD‑L1) + FLOT (fluorouracil, leucovorin, oxaliplatin, docetaxel) | pCR, MPR (TRG1a/b), R0 resection rate, ypT0, ypN0, surgical morbidity/mortality, Grade ≥3 TRAEs | pCR 24% vs 15% (FLOT), MPR 49% vs 38%, R0 resection ~96%; improved downstaging with similar safety profile |
| (Peng et al., 2025), RCT | China | 21 patients | Gastric adenocarcinoma (62%) and GEJ adenocarcinoma (21%) | Perioperative (3 cycles neoadjuvant + surgery + 5 cycles adjuvant) | Atezolizumab (anti–PD‑L1) + trastuzumab (HER2‑targeted) + XELOX (capecitabine + oxaliplatin) | pCR, MPR, ORR, R0 resection rate, EFS, DFS, OS, Grade ≥3 TRAEs, surgical complications | pCR 38.1% vs 14.3% (trastuzumab + XELOX); MPR 42.9% vs 19%; R0 resection ~95%; toxicity manageable, no new safety signals |
| (Shitara et al., 2025), RCT | Multinational (Asia, Europe, Americas) | 402 patients | Gastric adenocarcinoma (~79%) and GEJ adenocarcinoma (~21%) | Perioperative (3 cycles neoadjuvant + surgery + 3 cycles adjuvant chemo + 11 cycles adjuvant pembrolizumab) | Pembrolizumab (anti–PD‑1) + cisplatin/capecitabine (XP) or cisplatin/5‑FU (FP); separate FLOT cohort with pembrolizumab + FLOT | pCR, EFS, OS, R0 resection rate, surgical complications, Grade ≥3 TRAEs, patient‑reported outcomes | pCR 13.4% vs 2.0% (main cohort), EFS HR 0.81, OS HR 0.86; improved pCR and EFS trend, OS not statistically significant, safety manageable |
| (Yuan et al., 2024), RCT | China | 54 patients | Gastric adenocarcinoma (≈66%) and GEJ adenocarcinoma (≈34%) | Perioperative (3 cycles neoadjuvant + surgery + 5 cycles adjuvant + toripalimab maintenance up to 6 months) | Toripalimab (anti–PD‑1) + SOX (S‑1 + oxaliplatin) or XELOX (capecitabine + oxaliplatin) | pCR, MPR (TRG0/1), R0 resection rate, surgical morbidity/mortality, Grade ≥3 TRAEs, irAEs | TRG0/1 44.4% vs 20.4%, pCR 22.2% vs 7.4%; improved pathological response with manageable toxicity and no excess surgical risk |

***Supplementary Figure 1. Subgroup meta-analysis of pathologic complete response (pCR) according to neoadjuvant regimen intensity (chemotherapy-only versus intensified regimens).***

***Supplementary Figure 2. Subgroup meta-analysis of major pathologic response (MPR) according to neoadjuvant regimen intensity (chemotherapy-only versus intensified regimens).***

***Supplementary Figure 3. Subgroup meta-analysis of pathologic complete response (pCR) according to study design.***

***Supplementary Figure 4. Subgroup meta-analysis of major pathologic response (MPR) according to study design.***

***Supplementary Figure 5. Subgroup meta-analysis of pCR according to treatment strategy.***

***Supplementary Figure 6. Subgroup meta-analysis of MPR according to treatment strategy.***

***Supplementary Figure 7. Leave-one-out sensitivity analysis for pooled pathologic complete response (pCR). Sequential exclusion of individual studies did not materially alter the pooled pCR estimate, supporting the robustness of the primary analysis.***

***Supplementary Figure 8. Leave-one-out sensitivity analysis for pooled major pathologic response (MPR). Sequential exclusion of individual studies did not materially alter the pooled MPR estimate, indicating that the overall result was not driven by any single study.***

**Table S1: Complete Search Strategies**

| **Database** | **Serach string** |
| --- | --- |
| **PubMed** | (("Stomach Neoplasms"[MeSH Terms] OR "gastric cancer*"[Title/Abstract] OR "stomach cancer*"[Title/Abstract] OR "gastric adenocarcinoma*"[Title/Abstract] OR "gastric carcinoma*"[Title/Abstract] OR "gastric neoplasm*"[Title/Abstract] OR "stomach neoplasm*"[Title/Abstract] OR "gastric tumor*"[Title/Abstract] OR "gastric tumour*"[Title/Abstract] OR "gastroesophageal junction cancer"[Title/Abstract] OR "gastro-oesophageal junction cancer"[Title/Abstract] OR "gastroesophageal junction adenocarcinoma"[Title/Abstract] OR "gastro-oesophageal junction adenocarcinoma"[Title/Abstract] OR "esophagogastric junction cancer"[Title/Abstract] OR "esophagogastric junction adenocarcinoma"[Title/Abstract] OR "gastroesophageal junction tumor*"[Title/Abstract] OR "esophagogastric junction tumor*"[Title/Abstract] OR "GEJ cancer"[Title/Abstract] OR "GEJ adenocarcinoma"[Title/Abstract] OR "EGJ cancer"[Title/Abstract] OR "EGJ adenocarcinoma"[Title/Abstract]) AND ("Neoadjuvant Therapy"[MeSH Terms] OR "neoadjuvant"[Title/Abstract] OR "preoperative"[Title/Abstract] OR "perioperative"[Title/Abstract] OR "induction therapy"[Title/Abstract]) AND ("Immune Checkpoint Inhibitors"[MeSH Terms] OR "Immunotherapy"[MeSH Terms] OR "Immunotherapy"[Title/Abstract] OR "immune checkpoint inhibitor*"[Title/Abstract] OR "checkpoint inhibitor*"[Title/Abstract] OR "PD-1"[Title/Abstract] OR "PD-L1"[Title/Abstract] OR "CTLA-4"[Title/Abstract] OR "programmed cell death protein 1"[Title/Abstract] OR "programmed death-ligand 1"[Title/Abstract] OR "programmed death-ligand 1"[Title/Abstract] OR "anti-PD-1"[Title/Abstract] OR "anti-PD-L1"[Title/Abstract] OR "pembrolizumab"[Title/Abstract] OR "nivolumab"[Title/Abstract] OR "sintilimab"[Title/Abstract] OR "camrelizumab"[Title/Abstract] OR "tislelizumab"[Title/Abstract] OR "toripalimab"[Title/Abstract] OR "atezolizumab"[Title/Abstract] OR "durvalumab"[Title/Abstract] OR "avelumab"[Title/Abstract] OR "ipilimumab"[Title/Abstract])) AND (2014:2026[pdat]) |
| **Embase** | ((gastric cancer* or stomach cancer* or gastric adenocarcinoma* or gastric carcinoma* or gastric neoplasm* or stomach neoplasm* or gastric tumor* or gastric tumour* or gastroesophageal junction cancer or gastro-oesophageal junction cancer or gastroesophageal junction adenocarcinoma or gastro-oesophageal junction adenocarcinoma or esophagogastric junction cancer or esophagogastric junction adenocarcinoma or GEJ or EGJ) and (neoadjuvant or preoperative or perioperative or "induction therapy") and (immunotherapy or PD-1 or PD-L1 or CTLA-4 or pembrolizumab or nivolumab or sintilimab or camrelizumab or tislelizumab or toripalimab or atezolizumab or durvalumab or avelumab or ipilimumab)).mp. |
| **Cochrane CENTRAL** | ("gastric cancer" OR "stomach cancer" OR "gastric adenocarcinoma" OR "gastric carcinoma" OR "gastric neoplasm*" OR "stomach neoplasm*" OR "gastric tumor*" OR "gastric tumour*" OR "gastroesophageal junction cancer" OR "gastro-oesophageal junction cancer" OR "gastroesophageal junction adenocarcinoma" OR "gastro-oesophageal junction adenocarcinoma" OR "esophagogastric junction cancer" OR "esophagogastric junction adenocarcinoma" OR "gastroesophageal junction tumor*" OR "esophagogastric junction tumor*" OR "GEJ cancer" OR "GEJ adenocarcinoma" OR "EGJ cancer" OR "EGJ adenocarcinoma")AND("neoadjuvant" OR "preoperative" OR "perioperative" OR "induction therapy")AND("immunotherapy" OR "immune checkpoint inhibitor*" OR "checkpoint inhibitor*" OR "PD-1" OR "PD-L1" OR "CTLA-4" OR "programmed cell death protein 1" OR "programmed death-ligand 1" OR "anti-PD-1" OR "anti-PD-L1" OR pembrolizumab OR nivolumab OR sintilimab OR camrelizumab OR tislelizumab OR toripalimab OR atezolizumab OR durvalumab OR avelumab OR ipilimumab) in Title Abstract Keyword - (Word variations have been searched) |
| **Web Of Science** | ("gastric cancer" OR "stomach cancer" OR "gastric adenocarcinoma" OR "gastric carcinoma" OR "gastric neoplasm*" OR "stomach neoplasm*" OR "gastric tumor*" OR "gastric tumour*" OR "gastroesophageal junction cancer" OR "gastro-oesophageal junction cancer" OR "gastroesophageal junction adenocarcinoma" OR "gastro-oesophageal junction adenocarcinoma" OR "esophagogastric junction cancer" OR "esophagogastric junction adenocarcinoma" OR "gastroesophageal junction tumor*" OR "esophagogastric junction tumor*" OR "GEJ cancer" OR "GEJ adenocarcinoma" OR "EGJ cancer" OR "EGJ adenocarcinoma")AND("neoadjuvant" OR "preoperative" OR "perioperative" OR "induction therapy")AND("immunotherapy" OR "immune checkpoint inhibitor*" OR "checkpoint inhibitor*" OR "PD-1" OR "PD-L1" OR "CTLA-4" OR "programmed cell death protein 1" OR "programmed death-ligand 1" OR "anti-PD-1" OR "anti-PD-L1" OR pembrolizumab OR nivolumab OR sintilimab OR camrelizumab OR tislelizumab OR toripalimab OR atezolizumab OR durvalumab OR avelumab OR ipilimumab) |
| **Scopus** | TITLE-ABS-KEY ( ( "gastric cancer" OR "stomach cancer" OR "gastric adenocarcinoma" OR "gastric carcinoma" OR "stomach neoplasm*" OR "gastric neoplasm*" OR "gastroesophageal junction cancer" OR "gastro-oesophageal junction cancer" OR "gastroesophageal junction adenocarcinoma" OR "gastro-oesophageal junction adenocarcinoma" OR "esophagogastric junction cancer" OR "esophagogastric junction adenocarcinoma" OR "GEJ cancer" OR "GEJ adenocarcinoma" OR "EGJ cancer" OR "EGJ adenocarcinoma" ) AND ( "neoadjuvant" OR "preoperative" OR "perioperative" OR "conversion therapy" OR "induction therapy" ) AND ( "immunotherapy" OR "immune checkpoint inhibitor*" OR "checkpoint inhibitor*" OR "PD-1" OR "PD-L1" OR "programmed cell death protein 1" OR "programmed death ligand 1" OR "anti-PD-1" OR "anti-PD-L1" OR pembrolizumab OR nivolumab OR sintilimab OR camrelizumab OR tislelizumab OR toripalimab OR atezolizumab OR durvalumab OR avelumab ) ) AND PUBYEAR > 2013 AND PUBYEAR < 2027 AND ( LIMIT-TO ( LANGUAGE , "English" ) ) |
